# Cu - Nitrogen doped graphene (Cu–N/Gr) nanocomposite as cathode catalyst in fuel cells – DFT study

**DOI:** 10.1016/j.heliyon.2023.e15989

**Published:** 2023-05-03

**Authors:** Yashas Balasooriya, Pubudu Samarasekara, Chee Ming Lim, Yuan-Fong Chou Chau, Muhammad Raziq Rahimi Kooh, Roshan Thotagamuge

**Affiliations:** a Postgraduate Institute of Science, University of Peradeniya, Peradeniya, Sri Lanka; b Department of Physics, Faculty of Science, University of Peradeniya, Peradeniya, Sri Lanka; c Centre for Advanced Material and Energy Sciences, Universiti Brunei Darussalam, Jalan Tungku Link, Gadong BE 1410, Brunei Darussalam; d Department of Nano Science Technology, Faculty of Technology, Wayamba University of Sri Lanka, Kuliyapitiya 60200, Sri Lanka

**Keywords:** DFT, Fuel cell, Oxygen reduction reaction, Cathode catalyst, H_2_O_2_ generation

## Abstract

Novel Cu-nitrogen doped graphene nanocomposite catalysts are developed to investigate the Cu-nitrogen doped fuel cell cathode catalyst. Density functional theory calculations are performed using Gaussian 09w software to study the oxygen reduction reaction (ORR) on Cu-nitrogen doped graphene nanocomposite cathode catalyst in low-temperature fuel cells. Three different nanocomposite structures Cu_2_–N_6_/Gr, Cu_2_–N_8_/Gr and Cu–N_4_/Gr were considered in the acidic medium under standard conditions (298.15 K, 1 atm) in order to explore the properties of the fuel cell. The results showed that all structures are stable at the potential range 0–5.87 V. Formation energy, Mulliken charge and HOMO-LUMO energy calculations showed that Cu_2_–N_6_/Gr and Cu_2_–N_8_/Gr are more stable structure-wise, while free energy calculations showed that only Cu_2_–N_8_/Gr and Cu–N_4_/Gr structures support spontaneous ORR. The maximum cell potential under standard conditions was shown at 0.28 V and 0.49 V for Cu_2_–N_8_/Gr and Cu–N_4_/Gr respectively. From the calculations, the Cu_2_–N_6_/Gr and Cu_2_–N_8_/Gr structures are less favorable in H_2_O_2_ generation; however, Cu–N_4_/Gr showed the potential for H_2_O_2_ generation. In conclusion, Cu_2_–N_8_/Gr and Cu–N_4_/Gr are more favorable to ORR than Cu_2_–N_6_/Gr.

## Highlights

• Novel Cu_2_–N_6_/Gr, Cu_2_–N_8_/Gr motifs investigated.
• Cu_2_–N_6_/Gr, Cu_2_–N_8_/Gr are more stable than Cu–N_4_/Gr.
• Cu_2_–N_8_/Gr, Cu–N_4_/Gr showed spontaneous ORR.
• Inferior probability of H_2_O_2_ formation.
• Maximum cell potential reached 0.49 V.

## Graphical abstract

**Figure:**
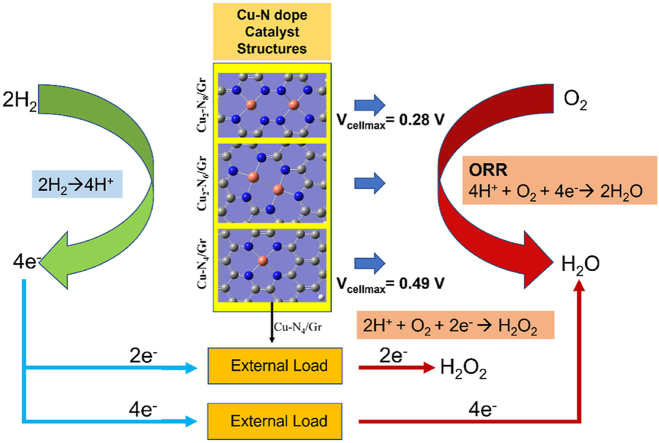

## 1 Introduction

Energy security and sustainability are the impetus for modern economics. Transformation of the energy ecosystem requires the search for cost-effective, secure, and sustainable energy technologies to replace incumbent energy sources. Fuel cells contribute well to this transformation as they evidently have their merits, particularly for compactness and zero emissions, such as proton exchange membrane fuel cells (PEMFCs) [1].

Low-temperature fuel cells operate at temperatures below 200 °C and a carbon support catalyst is deployed to improve the oxygen reduction reaction (ORR). Carbon support catalysts are not conducive to high temperature operations, leading to an increased rate of carbon corrosion and, therefore, degrading the carbon support [2]. Corrosion of the carbon support will directly affect the catalyst layer, causing the catalyst materials to agglomerate.

Generally, low-temperature commercial fuel cells use noble metals, such as Pt and Pd, as a catalyst. These noble metal catalysts can efficiently adsorb and split hydrogen into hydrogen ions [3,4] at the anode and provide a better ORR at the cathode [5]. The damage to the catalyst via carbon corrosion, where the carbon support starts to oxidize, the catalyst particles coated on top of the carbon support then lose their support and start to agglomerate, hence, resulting in diminished fuel cell performance over a short operation period.

To reduce the cost due to carbon corrosion, cheaper alternatives are used to replace the catalyst layer. One such example is the non-platinum group metal (non-PGM) catalysts. Non-PGM catalysts are usually produced using nitrogen atoms doped onto a graphite layer [[6], [7], [8]] or a layer of transition metals [9]. These are easy to produce and are much less expensive than PGM or PGM alloy catalysts such as Pt/Ni/Ir and Pt/Ni [[10], [11], [12]]. In addition, non-PGM have better electron conductivity. Fe/N/Gr, Co/N/Gr, and Mn/N/Gr are the most commonly used dopant [13,14] and are studied. The Mn/N/Gr type catalyst has a more stable potential cycle than Fe; however, the catalyst is not stable after the cathode potential exceeds 0.53 V [15]. Furthermore, the Mn–N_4_/Gr catalyst has OOH dissociation at 0.38 eV [15].

For Co-based non-PGM catalysts, Co–N_4_ has electrode stability between 0 and 1.23 V, and Co–N_2_ is only stable below 0.45 V [16]. Furthermore, its OOH binding energy is −1.02 eV for Co–N_4_ and −1.75 eV for Co–N_2_ without solvent, which are more favorable than Mn. Even with the best stability of the Co–N_4_ catalyst, research showed that Co–N_2_ has a stronger trend towards the ORR two-electron pathway and produces H_2_O_2_ as an intermediate [16]. H_2_O_2_ causes degeneration of the electrolyte membrane.

Compared to Mn and Co, Fe-based non-PGM catalysts produce less H_2_O_2_ and this H_2_O_2_ generation can be reduced by increasing the Fe content to 1.0 wt %. The generation of H_2_O_2_ in the ORR process at 0.1 wt % for common transition metals is as follows [17], N/Gr (39.4%) > N/Gr/Ni (39.0%) > N/Gr/Mn (23.9%) > N/Gr/Co (16.8%) > N/Gr/Fe (9.6%).

The formation of Fe(OH)_2_ during the ORR process [18] can remove Fe atoms from the carbon layer, which reduces the ORR efficiency, and the N/Gr/Fe catalyst can degenerate by oxidation [19].

Research on copper is mainly based on a single metal with nitrogen atoms as dopants [20,21], which gives good ORR performance, and spontaneous ORR makes them more favorable for catalysts. A single metal Cu–N_2_/Gr structure shows that it has a higher probability of following two-electron pathways to generate H_2_O_2_. In addition, there was no indication of the cathode potential of the Cu-nitrogen doped catalyst. A single structure of Cu–N_2_/Gr cannot describe the H_2_O_2_ generation capability or the cell potential of the Cu-nitrogen doped catalyst. Therefore, other structures, such as dual-metal structures, should be considered. Research shows that Fe-based non-PGM catalysts Fe_2_–N_6_/Gr and Fe_2_–N_8_/Gr have good formation energies and ORR performance [22,23]. Since Cu has shown better ORR performance steps than Fe [24], the Cu_2_–N_6_/Gr and Cu_2_–N_8_/Gr structures should have considerably better ORR performance than the single metal Cu-nitrogen doped catalyst or Fe-based non-PGM catalysts. Nørskov et al. reported that Cu as a dopant with nitrogen in graphite gives ORR performance almost as Pt (111) [24] catalyst surface for the binding energy of O and OH. Furthermore, Cu shows a good binding energy of OH and OOH, indicating stable catalysts for ORR [25,26].

The DFT method is an effective technique to study ORR performance and catalyst properties [27]. Therefore, in this study, DFT calculations were performed to determine the ORR mechanism and stability of Cu_2_–N_6_/Gr, Cu_2_–N_8_/Gr, and Cu–N_4_/Gr electrocatalysts in an acidic medium with different potentials at 298.15 K. From these calculations, the structural stability, the binding ability of ORR steps, the break-free ability of H_2_O, evidence of H_2_O_2_ generation, and the maximum cell potential were predicted.

## 2 Methodology

DFT calculations were performed with Gaussian 09w software using the B3LYP/3-21G basis set with non-periodic and non-dispersion interaction as Bhatt et al. [21]. Defect structure is visualized using GaussView 6.0. All atoms in every structure were relaxed by optimization. A common single pristine graphene layer was developed, and it is used to develop other structures (Cu_2_–N_6_/Gr, Cu_2_–N_8_/Gr, and Cu–N_4_/Gr) to investigate ORR considering the defect structure at the catalyst surface. All structures were developed to contain only pyridinic nitrogen. After optimization, zero imaginary frequency for dual atom structures and one (1) imaginary frequency for the Cu–N_4_/Gr structure. Additionally, the optimized charges of all three catalysts were zero and singlet spin for all dual atom structures. The molecules used in the ORR steps were individually optimized. Bond lengths and bond angles were measured and compared with those of previous research.

Catalyst structures based on Cu confirmed their stability with the cathode potential. Stability was inspected using the formation energy (Δ*E*) defined accordingly in the Eq. (1) [16,21],

(1) $$\Delta E=E_{graphene+\left(M_{a}-N_{b}\right)}+y\mu_{C}-\left(E_{graphene}+x\mu_{N}+E_{M}\right)$$

Here, $E_{graphene+\left(M_{a}-N_{b}\right)}$ is energy of optimized graphene layer with Cu–N defect. The ‘*a*’ and ‘*b*’ are positive integers defining the selected Cu–N defects (a = 1,2 and b = 4,6,8). M and N are Cu and nitrogen, respectively. The $\mu_{C}$ and $\mu_{N}$ are chemical potential of carbon defined as total energy per carbon atom for defect-free graphene, and the chemical potential of nitrogen defined as half of the total energy of N_2_ molecule, respectively. x and y are the number of nitrogen atoms added and the carbon atoms removed during the defect formation, respectively. $E_{graphene}$ is the energy of optimized pristine graphene layer. $E_{M}$ is the total energy of M^n +^ defined as Eq. (2),

(2) $$E\left(M^{n+}\right)=E\left(M\right)-neU$$

where, E(M) is the total energy of isolated M (M = Cu) in the gas phase and, *n*, *e*, *U* are the number of electron transfer (+2), electron charge, and external potential in order.

Binding energies (BE) were calculated from the ORR steps in an acidic medium, as defined by Eq. (3) [16,20,21],

(3) $$BE=E_{defect+molecule}-\left(E_{defect}+E_{molecule}\right)$$

Here, $E_{defect+molecule}$ is the total energy of molecules adsorbed by defect graphene. $E_{defect}$ is the total energy of defect graphene configuration, and $E_{molecule}$ is isolated molecule species (O_2_, O, H_2_O, OOH, OH, H_2_O_2_). Negative signed binding energies ($E_{defect+molecule}<\left(E_{defect}+E_{molecule}\right)$) indicate it is more favorable for molecules to be attached to the defect configuration. The formation of H_2_O_2_ during ORR is also considered.

ORR steps in H^+^ medium are defined as follows [16], * indicates the configuration of the defect.

* + O_2_ + 4H^+^ + 4e^−^ → *O_2_ + 4H^+^ + 4e^−^

*O_2_ + 4H^+^ + 4e^−^ → *OOH + 3H^+^ + 3e^−^

*OOH + 3H^+^ + 3e^−^ → *O + H_2_O + 2H^+^ + 2e^−^

*O + H_2_O + 2H^+^+ 2e^−^ → *OH + H_2_O + H^+^ + e^−^

*OH + H_2_O + H^+^ + e^−^ → 2H_2_O

The free energies were calculated for each ORR step of all defects configurations as defined by Eq. (4) [16],

(4) $$\Delta G=\Delta E+\Delta ZPE-T\Delta S+\Delta G_{U}+\Delta G_{pH}+\Delta G_{field}$$

ΔE is the energy from the DFT calculation to the relevant reaction step, and ΔZPE is the correction of zero-point energy is obtained from the NIST database and DFT calculations. *T* and *S* are the absolute temperature and entropy, respectively. Here, *T* = 298.15 K*,* and the entropy value was obtained from the NIST database. *ΔG*_*U*_ = *-eU* where *U* and *e* are the electrode potential and the charge transferred, respectively. $\Delta G_{pH}=K_{B}T\times \ln\;10\times pH$, where *K*_*B*_ is Boltzmann's constant and T = 298.15 K $\Delta G_{field}$ is a contribution of the interaction of adsorbate with the local electric field in the electric double layer formed in the vicinity of the cathode, which is negligible according to a Nørskov et al. [24]. Corrections of zero-point energy and entropy values were obtained from frequency calculations for the molecule adsorb by defect graphene. The free energy *vs*. reaction coordinate graphs were drawn with different potentials (*U*) for each structure. Additionally, the free energy of O_2_, 4.92 eV, was obtained from the literature under standard conditions with reaction O_2_ + 2H_2_ → 2H_2_O [24,28].

The Mulliken charge population was applied to molecules as defined by Eq. (5) [29],

(5) ΔQ_x_ = Q_after_ – Q_before_

The Q_after_ and Q_before_ are charges of the molecule (X) after and before adsorption, respectively.

The energy gap (E_g_), chemical hardness(η), chemical potential (μ), and electrophilicity index (ω) were calculated using the Koopman's principle for optimized structures as defined by Eqs. (6), (7), (8), (9), respectively [[30], [31], [32], [33]],

(6) $$E_{g}=E_{LUMO}-E_{HOMO}$$

(7) η=I−A

(8) $$\mu =-\frac{\left(I+A\right)}{2}$$

(9) $$\omega =\frac{\mu^{2}}{2\eta }$$

Here, *I* and *A* are the ionization potential (≅−E_HOMO_) and the electron affinity (≅−E_LUMO_), respectively.

## 3 Results and discussion

### 3.1 Formation energy

The formation energies of the Cu_2_–N_6_/Gr, Cu_2_–N_8_/Gr, and Cu–N_4_/Gr structures were −23.60, −23.49 and −13.22 eV in order at zero potential (U = 0). These values were only applied under open circuit conditions, and they changed with different cathode potentials according to Eq. (1) [16,21]. Table 1 shows that the formation energy increased with increasing external potentials. A negative sign means energy value gain when a structure forms. Therefore, the negative formation energies are favorable for the formation of stable structures, and the formation energies of all the structures studied remain negative between the potential range of 0–5.87 V. The critical cell potential of negative formation energies was observed at 5.89, 5.87, and 6.60 V for Cu_2_–N_6_/Gr, Cu_2_–N_8_/Gr, and Cu–N_4_/Gr, respectively, see Table 1. The critical *U* value at zero formation energy demarcates the stability/instability of the cathode. The structures forming stability follow the increasing order of Cu–N_4_/Gr < Cu_2_–N_8_/Gr < Cu_2_–N_6_/Gr, as indicated by the external potential, *U*, at zero.

**Table 1:** Formation energies for Cu_2_–N_6_/Gr, Cu_2_–N_8_/Gr, and Cu–N_4_/Gr structures with different external potentials (*U*).

Defect	External potential (*U*)/V	Formation energy/eV	Structure
CU_2_–N_6_/GR	0.00	−23.60	
	1.00	−19.60	
	2.00	−15.60	
	5.89	−0.04	
	*5.90*	*0.00*	
CU_2_–N_8_/GR	0.00	−23.49	
	1.00	−19.49	
	3.00	−11.49	
	5.87	−0.01	
	*5.88*	*0.03*	
CU-N_4_/GR	0.00	−13.22	
	1.00	−11.22	
	4.00	−5.22	
	6.60	−0.02	
	*6.61*	*0.00*	

### 3.2 Binding ability

The binding energies of ORR intermediates were calculated in an acidic medium involving O_2_, O, H_2_O, OOH, OH and H_2_O_2_ molecules (Table 2), and with and without the water solvent energies obtained from the work of Sha et al. [34]. Kattel et al. and Bhatt et al. reported that the binding energies of O_2_ to the structure of Co–N_4_/Gr and Cu–N_2_/Gr were −0.67 and −2.90 eV, respectively. As a first ORR step, O_2_ binding to the defect shown in this study is significantly high, except for the Cu–N_4_/Gr structure in relation to Cu–N_2_/Gr [16,21]. Structure-wise Cu–N_2_/Gr has a higher O_2_ binding energy than Cu–N_4_/Gr, also, Cu_2_–N_6_/Gr has a higher binding energy compared to the Cu_2_–N_8_/Gr structure. This indicates that adding more nitrogen atoms to Cu did not create a good O_2_ adsorbent defect. The efficiency of the O_2_ adsorbent in decreasing order is as follows, Cu_2_–N_6_/Gr > Cu_2_–N_8_/Gr > Cu–N_4_/Gr.

**Table 2:** Binding energies (BE) of Cu_2_–N_6_/Gr, Cu_2_–N_8_/Gr, Cu–N_4_/Gr structures with and without solvent energies (SE) [34] and shortest distance (d) between Cu–O, O–O atoms in angstroms (Å). The Mulliken charge of the adsorbate (Q_x_) and the Mulliken charge of the Cu site (Q_Cu_) for the shortest distance of the optimized structure. (01) and (02) in the Cu_2_–N_6_/Gr defect represent the different ORR paths 03-04-06-08 and 01-05-08 in Fig. 1, respectively. Two Mulliken charge values represent the atom order of the molecule column. Here, e = 1.602 × 10^−19^ C.

Defect	Molecule	BE (without solvent)/eV	SE/eV	BE (with solvent)/eV	d_O-O_/Å	d_CU-O_/Å	Q_x_/e	Q_Cu_/e
CU_2_–N_6_/GR (01)	O + O	−3.54	−0.41	−3.95	1.50	1.82	−0.223, −0.365	0.979
	OOH	−4.13	−0.47	−4.60	1.55	1.84	−0.328	0.934
	O	−7.53	−0.70	−8.23	–	1.74	−0.592	1.087
	OH	−5.12	−0.38	−5.50	–	1.89	−0.643	0.952
	H_2_O	−1.30	–	–	–	2.09	−0.573	0.937
	OH + OH _(H2O2)_	−5.44	–	–	2.48	1.75,1.85	−0.618, −0.602	0.947, 0.744
CU_2_–N_6_/GR (02)	O + O	−3.78	−0.41	−4.19	1.49	1.82	−0.367, −0.220	1.010
	O + OH	−9.52	−1.08	−10.60	2.52	1.83	−0.368, −0.661	0.631, 1.051
	OH + OH	−6.52	−0.76	−7.28	2.45	1.79	−0.665, −0.605	1.060, 0.897
	OH	−5.13	−0.38	−5.51	–	1.89	−0.640	0.949
	H_2_O	−1.30	–	–	–	2.09	−0.573	0.937
CU_2_–N_8_/GR	O_2_	−2.79	−0.41	−3.20	1.46	1.89	−0.273	0.967
	OOH	−3.27	−0.47	−3.74	1.55	1.93	−0.303	0.993
	OH + OH	−5.34	−0.76	−6.10	2.66	1.81,1.86	−0.606, −0.612	0.919,0.845
	OH	−3.81	−0.38	−4.19	–	2.05	−0.615	1.015
	H_2_O	−0.98	–	–	–	2.20	−0.599	1.068
CU-N_4_/GR	O_2_	−2.76	−0.41	−3.17	1.38	1.98	−0.195	0.996
	OOH	−1.70	−0.47	−2.17	1.52	1.87	−0.312	0.922
	O	−5.15	−0.70	−5.85	–	1.88	−0.312	0.905
	OH	−1.99	−0.38	–	–	1.84	−0.581	0.857
	H_2_O	−1.23	–	–	–	2.08	−0.587	1.011
	H_2_O_2_	−0.92	–	–	1.54	2.18	−0.319	0.999

The binding energies of OH and OOH on the Cu_2_–N_6_/Gr structure are shown to be −5.12 and −4.13 eV and −3.81 and −3.27 eV, respectively, for binding onto the Cu_2_–N_8_/Gr structure. The binding energies for OH and OOH to the Cu–N_2_/Gr structure were reported to be −2.68 and −1.81 eV, respectively, while for the Cu–N_4_/Gr structure were −1.99 and −1.70 eV [21]. The OH and OOH binding energies also signify the above N atoms and the idea of a stable defect. The energy values, shown in Table 2, also suggest that the Cu_2_–N_6_/Gr defect supports high ORR molecule binding.

**Fig. 1:**
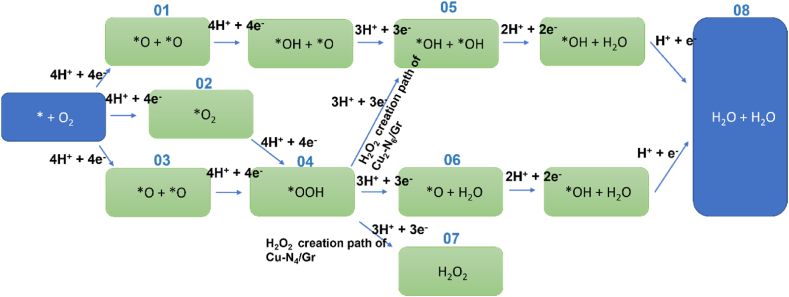
ORR paths for Cu_2_–N_6_/Gr, Cu_2_–N_8_/Gr, Cu–N_4_/Gr structures.

**Fig. 2:**
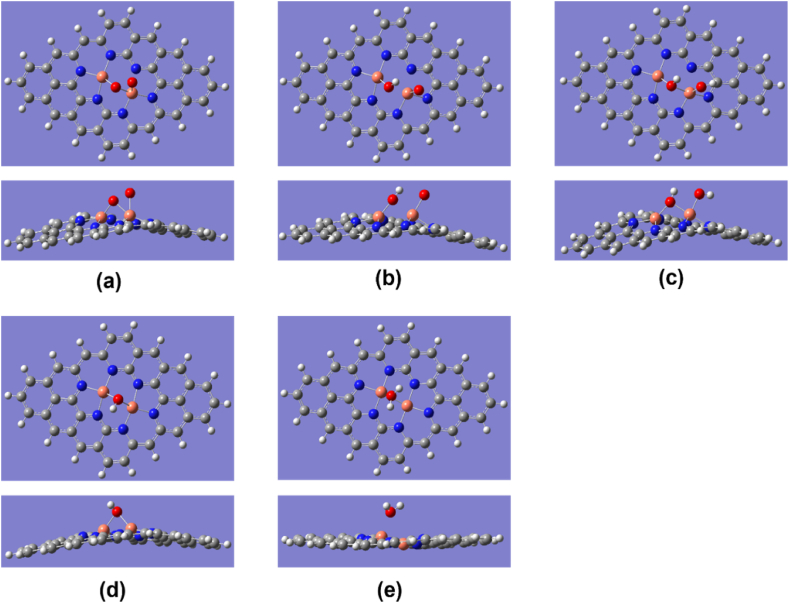
Top and side view of optimized ORR steps for Cu_2_–N_6_/Gr (02) structure. (a) O_2_ (b) O + OH (c) OH + OH (d) OH (e) H_2_O molecules binding on Cu_2_–N_6_/Gr (02) defect. (White, grey, blue, orange and red sphere represents hydrogen, carbon, nitrogen, copper and oxygen atoms, respectively). (For interpretation of the references to colour in this figure legend, the reader is referred to the Web version of this article.)

**Fig. 3:**
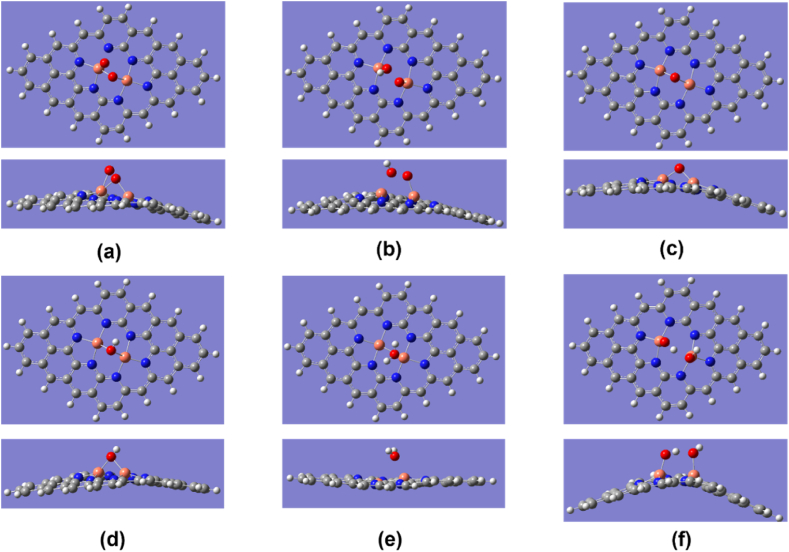
Top and side view of optimized ORR steps for Cu_2_–N_6_/Gr (01) structure. (a) O_2_ (b) OOH (c) O (d) OH (e) H_2_O (f) dual OH molecules form and binding instead of H_2_O_2_ on Cu_2_–N_6_/Gr (01) defect. (White, grey, blue, orange and red sphere represents hydrogen, carbon, nitrogen, copper and oxygen atoms, respectively). (For interpretation of the references to colour in this figure legend, the reader is referred to the Web version of this article.)

There are two possibilities of ORR pathways for Cu_2_–N_6_/Gr as shown in Fig. 1 and Table 2. Both pathways show that the O_2_ adsorbent is high relative to the other structures considered in this study. For the structure of Cu_2_–N_6_/Gr, the 1-5-8 pathway has a higher O_2_ adsorbent affinity than the 3-4-6-8 pathway. For both the 1-5-8 and 3-4-6-8 pathways, the initial step is the same, but having different binding energies may cause the ORR to take two different paths. Fig. 2(a)–(e) shows all five ORR steps of the 1-5-8 pathway with intermediates of *O_2_, *O + OH, *OH + OH, *OH, *H_2_O and Fig. 3(a)–(e) shows all five ORR steps of the 3-4-6-8 pathway with intermediates of *O_2_,*OOH, *O, *OH, *H_2_O respectively. Fig. 3(f) shows the result of trying to form H_2_O_2_ from the *OOH intermediate.

The difference in free energy (ΔG) for O_2_ binding was −3.59 and −3.58 eV for Cu_2_–N_6_/Gr (01) and Cu_2_–N_6_/Gr (02), respectively. These ΔG values indicate that the Cu_2_–N_6_/Gr structure favors both pathways, as shown by the O_2_ adsorption step.

**Fig. 4:**
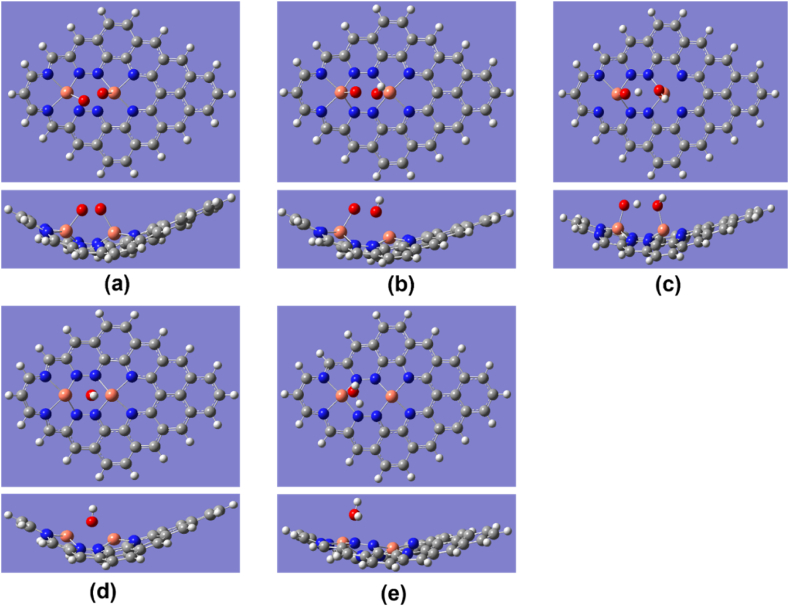
Top and side view of optimized ORR steps for Cu_2_–N_8_/Gr structure. (a) O_2_ (b) OOH (c) OH + OH (d) OH (e) H_2_O molecules binding on Cu_2_–N_8_/Gr defect. (White, grey, blue, orange and red sphere represents hydrogen, carbon, nitrogen, copper and oxygen atoms, respectively). (For interpretation of the references to colour in this figure legend, the reader is referred to the Web version of this article.)

The Cu–O bond length of O_2_ was 1.82, 1.89, and 1.98 Å for Cu_2_–N_6_/Gr, Cu_2_–N_8_/Gr, and Cu–N_4_/Gr, respectively. The results show that the most important first step of ORR favors the Cu_2_–N_6_/Gr structure. Xiao et al. and Isaacs et al. reported Cu–O bond lengths ranging from 2.04 to 2.59 Å [20,35]. However, in this study, the distance of Cu–O bond lengths is less than 2 Å for both the Cu_2_–N_6_/Gr and Cu_2_–N_8_/Gr structures, and this may be due to the binding of the O atom to dual Cu atoms as shown in Fig. 2, Fig. 3, Fig. 4. Here, Fig. 4(a)–(e) shows optimized ORR steps for Cu_2_–N_8_/Gr structure with *O_2_, *OOH, *OH + OH, *OH, *H_2_O intermediates respectively. For the Cu–N_4_/Gr structure, the Cu–O bond length is comparable to the minimum value of the Cu–O bond range studied previously. Furthermore, O–O bond length of O_2_ binding to the Cu–N_4_/Gr structure shows similarity to the Cu–N_2_/Gr structure [21]. Additionally, a previous investigation using VASP (Vienna ab-initio Simulation Package) [20] on Cu–N_4_/Gr indicated that the Cu–O bond lengths for the O_2_ and O intermediates were 2.22 Å and 1.67 Å, respectively, with O_2_ exhibiting weaker binding compared to O, potentially attributed to varying basis set configurations employed in the study. The O–O bond length of O_2_ binding in the Cu_2_–N_6_/Gr and Cu_2_–N_8_/Gr structures shows a slight increase in the bond length. This increase in the bond length may be due to π bonding electron on the adsorption side of O weakened by the Cu–O bond.

**Fig. 5:**
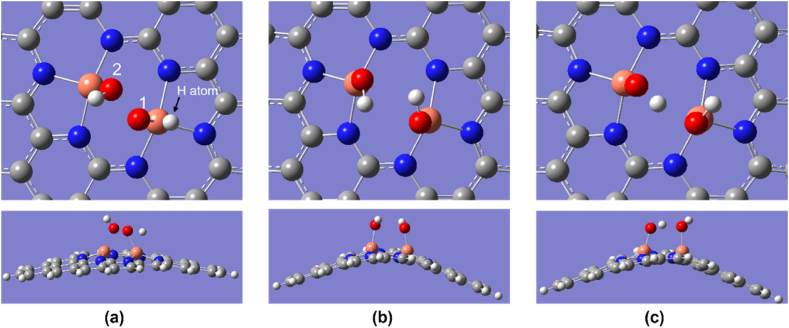
Cu_2_–N_6_/Gr (01) structure (a) H atom binds with O_1_ atom of OOH to form H_2_O_2_ (b) Middle state of optimization (c) Final optimization. (White, grey, blue, orange and red sphere represents hydrogen, carbon, nitrogen, copper and oxygen atoms, respectively). (For interpretation of the references to colour in this figure legend, the reader is referred to the Web version of this article.)

**Fig. 6:**
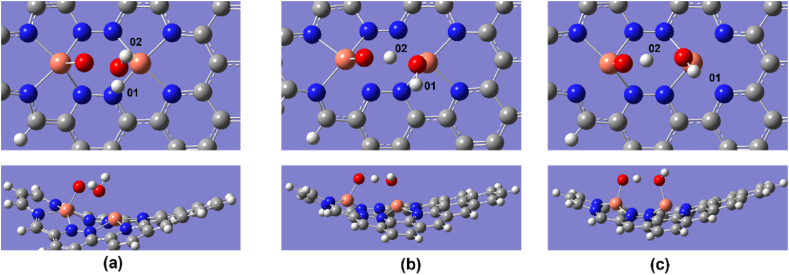
Cu_2_–N_8_/Gr structure (a) H atom (01) bind with second O atom of O_1_O_2_H molecule to form H_2_O (b) H atom (01) binding to second O atom and previously bonded H atom (02) break its bond and travel to first O atom (c) Final optimization, forming dual OH molecules instead of H_2_O_2_. (White, grey, blue, orange and red sphere represents hydrogen, carbon, nitrogen, copper and oxygen atoms, respectively). (For interpretation of the references to colour in this figure legend, the reader is referred to the Web version of this article.)

When molecule binding occurs, the Cu atom protrudes from the graphene layer. According to the optimized structures, most of the protruding Cu atom occur due to the binding of the O atom or the OH molecule (Fig. 3, Fig. 5, Fig. 6), and this is consistent with the work reported by Xiao et al. and Bhatt et al. [20,21]. The final ORR step involves the binding of H_2_O. The H_2_O binding strength follows a decreasing order with respect to the defect structures: Cu_2_–N_6_/Gr > Cu–N_4_/Gr > Cu_2_–N_8_/Gr. Based on the results of this study, it has been confirmed that the attachment of H_2_O to the Cu atom on the Cu_2_–N_8_/Gr defect structure is weakest, as evidenced by the 2.20 Å Cu–O bond length, when compared to the other defect structures investigated. On the other hand, Cu_2_–N_6_/Gr is shown to have a high H_2_O adsorbent possibility but also to have a better adsorption ability for O_2_ than H_2_O. The binding energies indicate that Cu–N_4_/Gr has less ability to adsorb H_2_O when compared to Cu_2_–N_6_/Gr. The bond length to H_2_O is similar for both defects. The binding energies of *OH, *OOH and *O have significantly higher values, indicating that all three defects could be considered as possible stable active sites for ORR.

### 3.3 Effects of the Mulliken charge

The Mulliken charge for the adsorbate of the optimized structures is shown in Table 2. The Mulliken charge population (ΔQ_x_) was applied to both O atoms of the O_2_ intermediate as Eq. (5) for all structures. ΔQ_x_ values for Cu_2_–N_6_/Gr (01) −0.063 e and 0.239 e, Cu_2_–N_6_/Gr (02) −0.141 e and −0.077 e, Cu_2_–N_8_/Gr 0.02 e and −0.151 e, Cu–N_4_/Gr −0.024 e. Here, the negative sign indicates an electron loss of the intermediate [29]. Therefore, the electron loss of the O atom could be considered as the electron gain of the Cu atom, which could explain the increased binding strength. The decreasing order of the charge population bonding strength is Cu_2_–N_6_/Gr > Cu_2_–N_8_/Gr > Cu–N_4_/Gr, suggesting that Cu_2_–N_6_/Gr has the highest O_2_ adsorption ability. Additionally, this observation is consistent with the decreasing order of the O_2_ adsorption efficiency predicted using the binding energy. For H_2_O, the Mulliken charge population values for are 0.027 e, 0.058 e, 0.008 e and −0.054 e for Cu_2_–N_6_/Gr (01), Cu_2_–N_6_/Gr (02), Cu_2_–N_8_/Gr and Cu–N_4_/Gr, respectively. These values signify the binding strength of H_2_O in decreasing order as Cu–N_4_/Gr > Cu_2_–N_8_/Gr > Cu_2_–N_6_/Gr (01) > Cu_2_–N_6_/Gr (02). Only Cu–N_4_/Gr and Cu_2_–N_8_/Gr are compared with the above H_2_O adsorbent strength decreasing order explained by the binding energy. The difference in H_2_O adsorbent strength order between the Mulliken charge population and the binding energy may be due to the O atom drawing electrons from H atoms of H_2_O, altering the Mulliken charge values of the O atom. Based on the Mulliken charges of Cu_2_–N_6_/Gr, it has the weakest bond strength on H_2_O, allowing H_2_O to break free from the catalyst easily. The Mulliken charges of the optimized structures in Table 2 indicate that the bonding capability is dependent on the difference of Q_x_ and Q_Cu_ and the bond length d_Cu-O_. Strong bonding is favored by a large difference in Q_x_ and Q_Cu_ and a short bond length d_Cu-O_.

The Cu_2_–N_6_/Gr structure follows the ORR steps on the 01-05-08 and 03-04-06-08 paths (Fig. 1). The 03-04-06-08 path has the possibility of generating H_2_O_2_ due to the formation of *OOH intermediate with an additional H atom (Fig. 5(a)), but optimization shows instead of H_2_O_2_ generation, it generates a separate *OH intermediates (Fig. 5(c)) and follows the 01-05-08 path from step 05 (Fig. 3, Fig. 5 and (f)).

Separate generation of the OH molecule may be due to the attraction of electrons by the Cu atoms. According to Mulliken charges, N atoms lose fewer electrons to Cu atoms of Cu_2_–N_6_/Gr on demand of electrons compared to Cu–N_4_/Gr. Therefore, the Cu atom of Cu_2_–N_6_/Gr may try to replenish its electron demand from O atom charges by forming a strong Cu–O bond (Fig. 5(b) and (c)) unlike Cu–N_4_/Gr which form H_2_O_2_. Furthermore, this scenario could be explained as π bonding electron on O atoms that attracts to each Cu atom. If the O atoms largely lose their electron charge density, that could cause the O–O bond to break and generate *2OH instead of H_2_O_2_ from *OOH.

Nallathambi et al. reported that H_2_O_2_ generation could stop the ORR four-electron path halfway [36]. The ORR steps of an acidic medium show that a two-electron pathway is needed for the generation of H_2_O_2_. However, the optimization did not produce a two-electron pathway for the Cu_2_–N_6_/Gr structure. Cu_2_–N_8_/Gr structures only follow the ORR 02-04-05-08 path (Fig. 1). It is a four-electron pathway without H_2_O_2_ generation. The *OOH intermediate on its ORR path is more favorable to form dual OH over H_2_O (Figs. 4(c) and 6(a)–(c)). Binding of the H atom (01) to the second O atom (Fig. 6(a)) causes the breakage of the O–O bond due to π electron deviation. This situation creates the H_2_O molecule short time. However, the formation of *2OH is made possible by the attraction of electrons from the O atom of H_2_O by the Cu atom caused by weak Cu–N bonding. This in turn would weaken one of the O–H bonds and lead to the release of H to react to form OH (Fig. 6(b)). Since the *OOH of Cu_2_–N_8_/Gr structure creates the *2OH configuration by H atoms binding to both O atoms (Fig. 6(c)), the possibility of H_2_O_2_ formation is reduced.

**Fig. 7:**
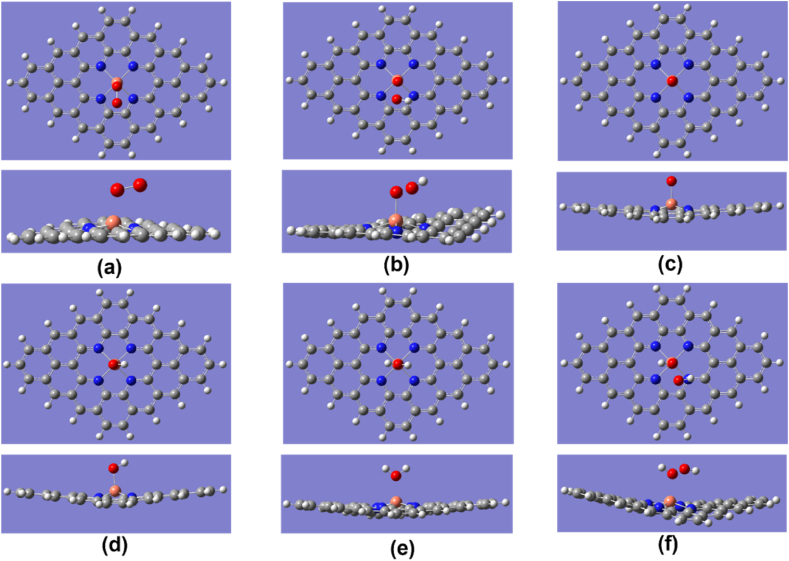
Top and side view of optimized ORR steps for Cu–N_4_/Gr structure. (a) O_2_ (b) OOH (c) O (d) OH (e) H_2_O (f) H_2_O_2_ molecules binding on Cu–N_4_/Gr defect. (White, grey, blue, orange and red sphere represents hydrogen, carbon, nitrogen, copper and oxygen atoms, respectively). (For interpretation of the references to colour in this figure legend, the reader is referred to the Web version of this article.)

The ORR associated with the Cu–N_4_/Gr structure follows only the 02-04-06-08 path (Fig. 1) with *O_2_, *OOH, *O, *OH, *H_2_O intermediates respectively (Fig. 7(a)–(e)). The optimized structures show the possibility of H_2_O_2_ generation (Fig. 7(f)). With a binding energy of −0.92 eV and a Cu–O bond length of 2.18 Å, H_2_O_2_ can easily break free from the Cu–N_4_/Gr defect (Fig. 7(f)). The O–O bond in the simulation is shown to be stable, as suggested by Bhatt et al. [21]. The Mulliken charge population value of H_2_O_2_, 0.026 e, indicates that H_2_O_2_ has the ability to break free from the catalyst surface as H_2_O. Since H_2_O_2_ directly participates in the degeneration of the fuel cell membrane [37,38], the structure of Cu–N_4_/Gr can significantly reduce the efficiency of the fuel cell. Additionally, the two-electron pathway of H_2_O_2_ generation can reduce the current density of the fuel cell.

### 3.4 HOMO and LUMO energy calculations

The HOMO and LUMO energies were calculated for every step of the ORR and structural defects to further investigate the binding energy. The energy gap (*E*_*g*_), chemical hardness (η), chemical potential (μ), and electrophilicity index (ω) of *O_2_, *H_2_O, *H_2_O_2,_ intermediates and defect structures were calculated as shown in Table 3 using Eqs. (6), (7), (8), (9)) [29,31,32].

**Table 3:** Energy gap (E_g_), chemical hardness (η), chemical potential (μ), and electrophilicity index (ω) values of *O_2_, *H_2_O, *H_2_O_2_ intermediates and defect structures.

Defect	Intermediate/structure	E_g_/eV	η/eV	μ/eV	ω/eV
CU_2_–N_6_/GR (01)	Cu_2_–N_6_/Gr	0.04	0.04	−0.13	0.23
	O + O	0.03	0.03	−0.14	0.33
	H_2_O	0.03	0.03	−0.12	0.23
	H_2_O_2_	–	–	–	–
CU_2_–N_6_/GR (02)	O + O	0.03	0.03	−0.14	0.35
	H_2_O	0.03	0.03	−0.12	0.22
	H_2_O_2_	–	–	–	–
CU_2_–N_8_/GR	Cu_2_–N_8_/Gr	0.05	0.05	−0.12	0.15
	O_2_	0.04	0.04	−0.13	0.19
	H_2_O	0.05	0.05	−0.11	0.13
	H_2_O_2_	–	–	–	–
CU-N_4_/GR	Cu–N_4_/Gr	0.03	0.03	−0.13	0.27
	O_2_	0.04	0.04	−0.13	0.19
	H_2_O	0.04	0.04	−0.11	0.16
	H_2_O_2_	0.03	0.03	−0.12	0.21

According to Eqs. (6), (7)) η and *E*_*g*_ are directly associated with each other. The chemical hardness elucidates the stability and reactivity of the structure. Therefore, stable structures are less reactive, and high-reactive structures are less stable [8,29,31]. Defects can be ranked in decreasing order of chemical hardness as Cu_2_–N_8_/Gr > Cu_2_–N_6_/Gr > Cu–N_4_/Gr. This suggests that the reactivity of the Cu–N_4_/Gr structure is higher than that of other structures, but it is also less stable. This reactivity could cause electrons in the valence band to be easily excited to occupy the conduction band. The reactivity order is confirmed by the formation energy shown in Table 1. The Cu_2_–N_8_/Gr structure is more stable than others and is the least reactive of the three defect structures, and Cu_2_–N_6_/Gr has medium structural stability and reactivity according to chemical hardness values.

The chemical hardness of the oxygen bonding decreasing order Cu_2_–N_8_/Gr = Cu–N_4_/Gr > Cu_2_–N_6_/Gr indicates that Cu_2_–N_6_/Gr is less stable and more reactive than the other two structures. If the ORR first step is less stable and has high reactivity, the probability of achieving the next ORR step is high. Therefore, the Cu_2_–N_6_/Gr defect is more suitable to complete the ORR first step. From previous calculations, the binding energy of the second step for ORR of Cu_2_–N_6_/Gr structure is higher than that of the first step, and this collaborates well with the chemical hardness data. The chemical hardness of the H_2_O bond in decreasing order is as follows: Cu_2_–N_8_/Gr > Cu–N_4_/Gr > Cu_2_–N_6_/Gr. This indicates that the H_2_O bonded to the Cu_2_–N_8_/Gr defects is more stable than the other defects. DFT calculations also show that the longest bond length of Cu–O and the highest binding energy would increase the probability of breaking H_2_O from the Cu_2_–N_8_/Gr defect sites. In the case of H_2_O bonding to the highly reactive Cu_2_–N_6_/Gr defect, this would result in an oxygen evolution reaction (OER) [20,39].

The chemical potential is directly related to Mulliken's electronegativity [31]. The chemical reactivity of the structure increases with decreasing chemical potential. The chemical potential decreases in the following order of defects: Cu_2_–N_8_/Gr > Cu_2_–N_6_/Gr = Cu–N_4_/Gr. This shows that the chemical reactivity of the Cu_2_–N_6_/Gr and Cu–N_4_/Gr structures is slightly higher than that of Cu_2_–N_8_/Gr. Furthermore, the chemical reactivity of the oxygen-bonded structures is the highest for Cu_2_–N_6_/Gr, confirming the previous chemical hardness evaluation of Cu_2_–N_6_/Gr.

Since electrophilicity is the electron accepting capacity from the surrounding atoms to form a stable energy state, and this relates directly to the structural stability. The electrophilicity of the studied defects has the following decreasing order, Cu–N_4_/Gr > Cu_2_–N_6_/Gr > Cu_2_–N_8_/Gr, and this also confirms with the reactivity order of the structures in terms of chemical hardness. The oxygen-bonded Cu_2_–N_6_/Gr shows a higher electron acceptation than the other structures, hence, indicating that the Cu_2_–N_6_/Gr structure favors electron adsorption to form a stable structure. Two ORR steps of Cu_2_–N_6_/Gr that generate different paths could occur to this reactivity. The Cu_2_–N_8_/Gr and Cu–N_4_/Gr structures show similar electrophilicity values for the O_2_ bonding. The stability of the Cu–N_4_/Gr structure is attributed to the four nitrogen atoms that donate electrons to the Cu atom. Compared to the Cu_2_–N_6_/Gr structure, which has six nitrogen atoms, the Cu–N_4_/Gr structure is more stable (Table 1). The Cu_2_–N_8_/Gr structure has twice as many nitrogen atoms as Cu–N_4_/Gr, but its electrophilicity value is likely to be low, similar to that of Cu–N_4_/Gr. Furthermore, H_2_O-bonded Cu_2_–N_8_/Gr showed the lowest electrophilicity value for the last ORR step, indicating a low probability that a further reaction will take place.

### 3.5 Free energy

**Fig. 8:**
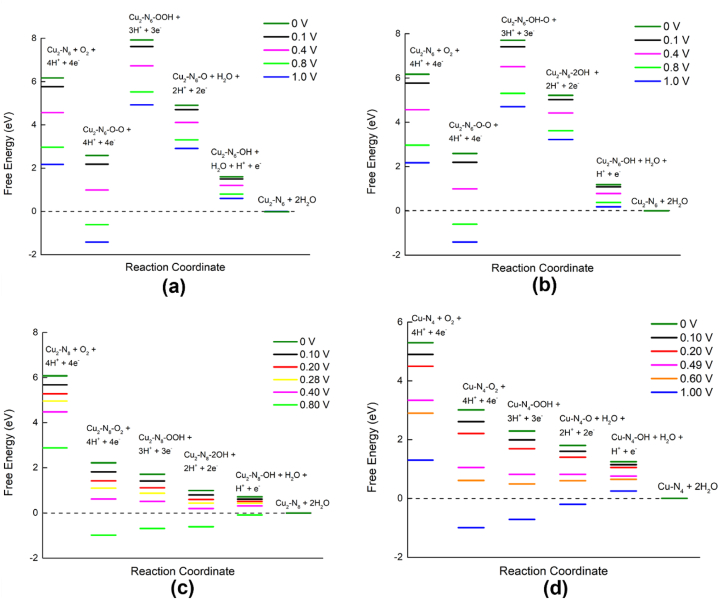
Free energy of possible ORR paths for Cu_2_–N_6_/Gr, Cu_2_–N_8_/Gr and Cu–N_4_/Gr defect structures with different potentials in acidic medium. (a) Cu_2_–N_6_/Gr (01) (b) Cu_2_–N_6_/Gr (02) (c) Cu_2_–N_8_/Gr (d) Cu–N_4_/Gr.

Free energy values were calculated for the three defect structures with possible ORR pathways in an acidic medium as shown in Fig. 8(a–d). Cu_2_–N_6_/Gr shows a spontaneous reaction (downhill) for the first two steps of ORR with cell potential (U) 0–1.00 V (Fig. 8(a) and (b)). The second to the third steps of the ORR for all potentials show uphill. From the third step to the last downhill, the configuration was found again for 0 V–1.00 V. This indicates that a large energy barrier is present between the second step and the third step, which prevents the spontaneous reaction of ORR from being completed. The highest reaction step barrier, 5.34 eV and 5.11 eV, are shown by Cu_2_–N_6_/Gr, for both (01) and (02) paths at U = 0 V, respectively, in *O–O hydrogenate by attaching the H atom to form *OOH and *O–OH. In summary, even with good binding energies, both ORR paths of Cu_2_–N_6_/Gr structure did not show spontaneity.

The Cu_2_–N_8_/Gr structure shows spontaneous reaction for all ORR steps for cell potential ranges from 0 to 0.28 V. With cell potential >0.28 V, the fifth step of ORR becomes uphill, showing the *2OH → *OH + H_2_O reaction process is not spontaneous (Fig. 8(c)). A further increase in the cell potential caused an increase in the uphill processes. The Cu–N_4_/Gr structure shows a spontaneous process for all ORR steps until the cell potential reaches 0.49 V. After the cell potential exceeds 0.49 V (*U* > 0.49 V), the ORR step of forming *O + H_2_O becomes uphill (Fig. 8(d)). Then, with the enhancement of the potential, the uphill process also increased. The maximum cell potentials for spontaneous reactions are shown to be 0.49 and 0.28 V for Cu–N_4_/Gr and Cu_2_–N_8_/Gr, respectively. These results show that the open circuit voltage (OCV) is relatively better than the Pt catalyst cathode, which initially shows 0.9 V, as reported by Kim et al. [40].

**Fig. 9:**
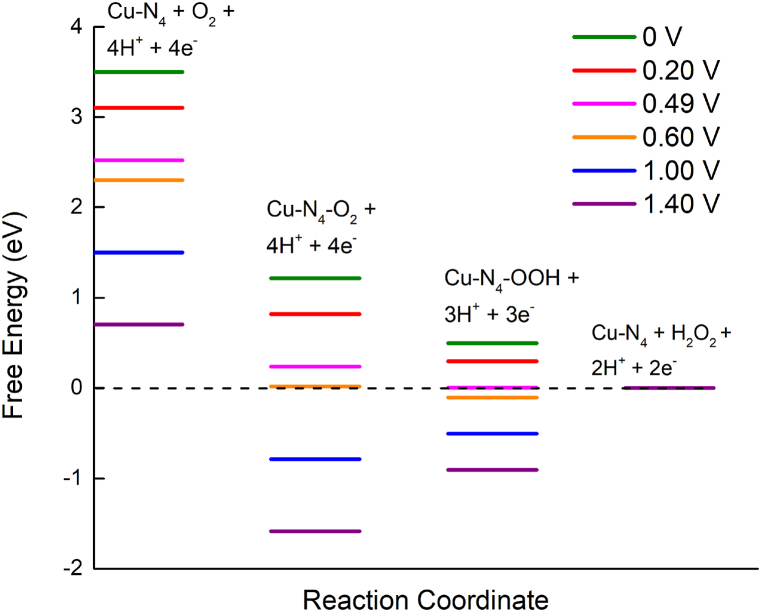
Free energy diagram of H_2_O_2_ generation in Cu–N_4_/Gr defect structures with different potentials in acidic medium.

A comparison of the formation energies and chemical hardness indicates that the Cu–N_4_/Gr structure has a lower probability of forming and is less stable than other structures. Therefore, generating 0.49 V as cell voltage is a low possibility. Furthermore, the Cu–N_4_/Gr defect structure has a greater possibility of forming H_2_O_2_, as indicated in Table 2. The free energy diagram (Fig. 9) shows that the formation of H_2_O_2_ was spontaneous within the range of 0–0.49 V. After exceeding 0.49 V, the H_2_O_2_ formation step becomes uphill. Both the free energy and the maximum potential of the downhill process to form H_2_O_2_ and H_2_O from *OOH are the same. Furthermore, the Mulliken charges of the O atoms of *OOH are approximately equal (−0.312 e and −0.331 e, respectively), indicating that the final step of ORR of Cu–N_4_/Gr is solely dependent on the binding of the H atom to the O atom of *OOH (Fig. 7(b)). If the H atom binds to the Cu–O side O atom of *OOH, it could generate H_2_O_2,_ and binding to the other O atom would generate 2H_2_O in the final step. Since the H_2_O_2_ formation path supplies only two electrons through an external path, from the anode to the cathode, this could result in a decrease in current density.

However, this study has several limitations that should be acknowledged. First, the 3-21G basis set used in this study can be considered small. However, it was selected based on Bhatt et al.'s study [21], which compared Cu–N_2_/Gr results with the current study. Furthermore, this study did not include periodic boundary conditions or dispersion interactions, unlike the work by Xiao et al. [20] on Cu–N_4_/Gr. However, these studies cannot be used as a basis for a comparison with the current study due to their use of different DFT simulation software. The use of Gaussian09 instead of VASP-like simulation software was due to financial constraints. Although VASP-like software is better suited for surface simulation and PBC approach calculations, this study employed a single layer of graphene to examine the impact of defect structures on the surface of the catalyst in relation to ORR, similar to the approaches taken by Bhatt et al. [21] and Kattel et al. [16]. Future studies related to this research will employ dispersion correction with the PBC approach to provide a basis for comparison with the current study.

## 4 Conclusion

Computational calculations predicted the Cu_2_–N_8_/Gr and Cu–N_4_/Gr motifs, showing promising spontaneous ORR with maximum cell potential of 0.28 and 0.49 V under standard conditions. The Cu_2_–N_6_/Gr motif is not favorable for spontaneous ORR. From the formation energy calculations, Cu_2_–N_8_/Gr, Cu–N_4_/Gr, and Cu_2_–N_6_/Gr are stable for potential range (U) 0–5.87, 0–6.6, and 0–5.89 V, respectively. The maximum cell potentials of Cu_2_–N_8_/Gr and Cu–N_4_/Gr are within the potential range of formation energy, showing structural stability. Based on the chemical hardness, chemical potential, electrophilicity, and Mulliken charge calculations, the Cu_2_–N_8_/Gr shows a higher stability and favors ORR than Cu_2_–N_6_/Gr. Cu_2_–N_6_/Gr is more favorable for ORR than Cu–N_4_/Gr by binding energy values. The Cu_2_–N_6_/Gr motif is not recognized as a spontaneous catalyst by free energy calculations. Therefore, considering these three motifs, only Cu_2_–N_8_/Gr and Cu–N_4_/Gr show promising ORR ability. Consequently, the possibility of cell voltage solely depends on Cu_2_–N_8_/Gr could occur due to its high stability. According to DFT optimization, H_2_O_2_ is generated only from Cu–N_4_/Gr. The other structures follow four-electron transfer pathways to generate high electron density. The Cu-Nitrogen doped non-PGM catalyst with the considered motifs has a relatively high current density even with low cell voltage when comparing to Pt-like catalysts. The amount of H_2_O_2_ generated by Cu–N_4_/Gr could be limited by its low stability. Bhatt et al. have also shown that the Cu–N_2_/Gr motif has a high probability of generating H_2_O_2,_ but the −5.68 eV formation energy [21] indicates that it has lower stability than Cu–N_4_/Gr. In this study, a novel Cu_2_–N_8_/Gr catalyst is a good candidate for fuel cell application as it has good cell potential and non-H_2_O_2_ formation. Furthermore, the three structures presented in this study show a better O_2_ binding energy than the Pt catalyst and PGM alloys [41].

## Author contribution statement

Yashas Balasooriya: Conceived and designed the experiments; Performed the experiments; Analyzed and interpreted the data; Wrote the paper.

Pubudu Samarasekara, Muhammad Raziq Rahimi Kooh: Conceived and designed the experiments; Contributed reagents, materials, analysis tools or data; Wrote the paper.

Chee Ming Lim, Yuan-Fong Chou Chau: Contributed reagents, materials, analysis tools or data; Wrote the paper.

Roshan Thotagamuge: Conceived and designed the experiments; Analyzed and interpreted the data; Contributed reagents, materials, analysis tools or data; Wrote the paper.

## Data availability statement

Data included in article/supplementary material/referenced in article.

## Funding

The work described in this article was supported by the 10.13039/100009100Universiti Brunei Darussalam Research Grant 10.13039/100009100UBD/RSCH/1.9/FICBF(b)/2022/017.

## Declaration of competing interest

The authors declare that they have no known competing financial interests or personal relationships that could have appeared to influence the work reported in this paper.
