# Supplementary material for: Cu - Nitrogen doped graphene (Cu–N/Gr) nanocomposite as cathode catalyst in fuel cells – DFT study
Source: Heliyon. 2023 May 3;9(5):e15989. doi: 10.1016/j.heliyon.2023.e15989 (PMC10195903; doi:10.1016/j.heliyon.2023.e15989)
Supplement: Multimedia component 1 [file mmc1.docx]

**Appendix A. Supplementary data**

Cartesian coordinates of catalyst structures according to figures in manuscript.

Cu2-N6/Gr

| Symbol | X | Y | Z |  |
| --- | --- | --- | --- | --- |
| C | -5.87692 | -1.09273 | -0.01008 | 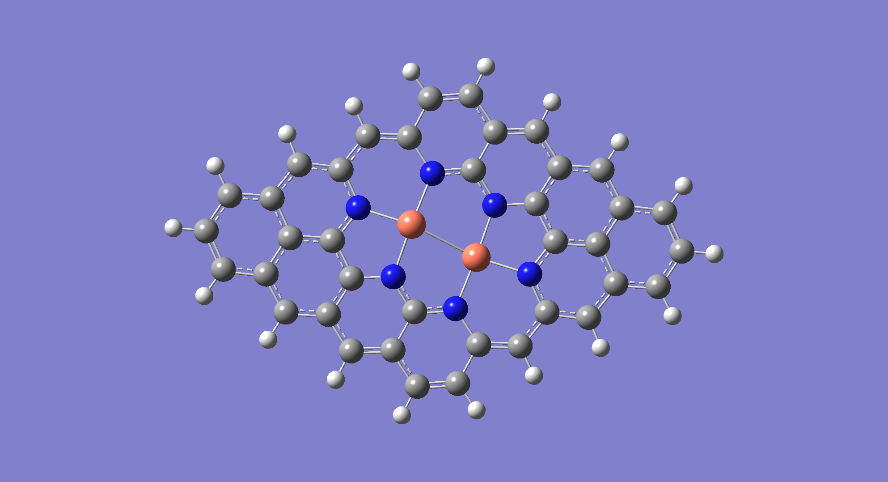 |
| C | -5.08136 | 0.105966 | -0.00328 |  |
| C | -3.68329 | 0.017642 | 0.001129 |  |
| C | -3.05698 | -1.22862 | 0.036658 |  |
| C | -3.82154 | -2.4219 | -0.00239 |  |
| C | -5.22218 | -2.34528 | -0.01839 |  |
| C | 0.976416 | 2.333811 | -0.05253 |  |
| C | 3.68328 | -0.01764 | -0.00085 |  |
| C | 3.402462 | -2.35117 | 0.007761 |  |
| C | 5.081356 | -0.10597 | 0.003626 |  |
| C | 4.782028 | -2.52077 | 0.005313 |  |
| C | 2.515725 | -3.4672 | 0.006445 |  |
| C | 5.876916 | 1.092726 | 0.010274 |  |
| C | 5.680588 | -1.40989 | 0.016178 |  |
| C | 7.292089 | 0.931937 | 0.029931 |  |
| C | 7.078377 | -1.50222 | 0.033258 |  |
| C | 7.859064 | -0.33221 | 0.037613 |  |
| C | 1.146603 | -3.42294 | 0.015461 |  |
| C | -3.06395 | -3.62605 | -0.00058 |  |
| C | -1.68843 | -3.59697 | 0.015916 |  |
| C | -0.97642 | -2.33382 | 0.052048 |  |
| C | -0.88865 | -4.79519 | -0.00583 |  |
| C | 0.453061 | -4.69967 | 0.002522 |  |
| C | 3.056987 | 1.228624 | -0.03673 |  |
| C | 5.222173 | 2.345293 | 0.018277 |  |
| C | 3.821546 | 2.421919 | 0.002126 |  |
| C | 3.063942 | 3.62607 | -9.8E-05 |  |
| C | 1.688432 | 3.596971 | -0.01671 |  |
| H | 7.553664 | -2.47599 | 0.043699 |  |
| H | 5.18119 | -3.52734 | 0.030873 |  |
| H | 2.974799 | -4.44743 | -0.01254 |  |
| H | 1.075708 | -5.58476 | -0.00786 |  |
| H | 7.920254 | 1.81513 | 0.049686 |  |
| H | 8.939494 | -0.42576 | 0.05349 |  |
| C | -5.68061 | 1.409902 | -0.01564 |  |
| C | -4.78204 | 2.520784 | -0.00487 |  |
| C | -7.07839 | 1.502223 | -0.03257 |  |
| C | -3.40248 | 2.351174 | -0.00739 |  |
| C | -2.51572 | 3.467185 | -0.00646 |  |
| C | -1.14661 | 3.422908 | -0.01598 |  |
| C | -0.45307 | 4.699637 | -0.0033 |  |
| C | 0.888644 | 4.795186 | 0.004825 |  |
| C | -7.29211 | -0.93193 | -0.02956 |  |
| C | -7.85908 | 0.332206 | -0.03699 |  |
| H | -7.55368 | 2.475994 | -0.04284 |  |
| H | -5.1812 | 3.527352 | -0.03052 |  |
| H | -2.97477 | 4.447426 | 0.012541 |  |
| H | -1.07573 | 5.584723 | 0.007038 |  |
| H | 1.391287 | 5.754744 | 0.026101 |  |
| H | 3.575831 | 4.58234 | 0.022994 |  |
| H | 5.808488 | 3.256822 | 0.026664 |  |
| H | -1.39131 | -5.75473 | -0.02732 |  |
| H | -3.57585 | -4.58231 | -0.02386 |  |
| H | -5.80848 | -3.25682 | -0.02692 |  |
| H | -7.92027 | -1.81513 | -0.04939 |  |
| H | -8.93951 | 0.425765 | -0.05274 |  |
| N | -0.38172 | 2.234324 | -0.03159 |  |
| N | -2.83375 | 1.094671 | -0.04639 |  |
| N | -1.67621 | -1.18062 | 0.116077 |  |
| N | 0.381723 | -2.23439 | 0.031157 |  |
| N | 2.833734 | -1.09465 | 0.046936 |  |
| N | 1.67626 | 1.180616 | -0.11635 |  |
| Cu | -1.0697 | 0.544993 | 0.216162 |  |
| Cu | 1.069714 | -0.54498 | -0.21591 |  |

Cu2-N8/Gr

| Symbol | X | Y | Z |  |
| --- | --- | --- | --- | --- |
| C | -5.65065 | 1.249054 | -0.57503 | 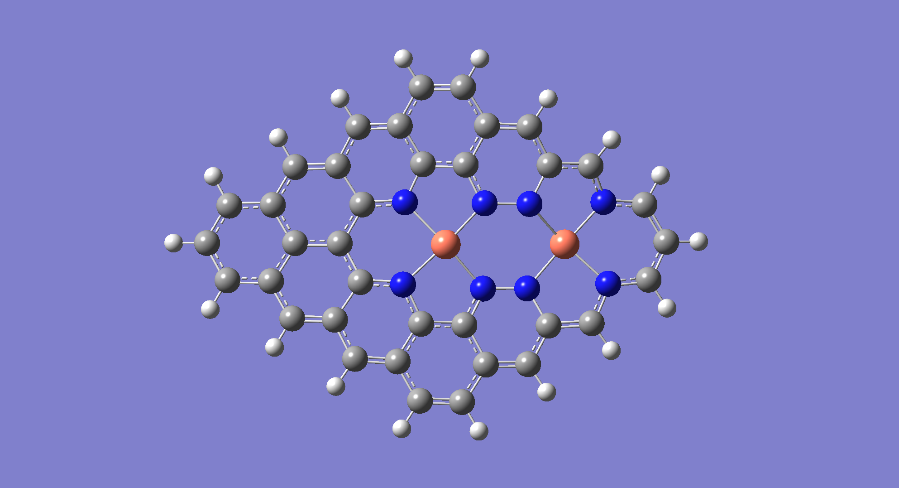 |
| C | -4.93879 | 0.028065 | -0.31279 |  |
| C | -3.5097 | 0.021255 | -0.12519 |  |
| C | -2.79081 | 1.263116 | -0.18861 |  |
| C | -3.5726 | 2.493122 | -0.27827 |  |
| C | -4.9328 | 2.460139 | -0.51388 |  |
| C | -2.84783 | -1.2159 | 0.168734 |  |
| C | -0.90731 | -2.54966 | 0.351787 |  |
| C | 0.475064 | -2.59157 | 0.147208 |  |
| C | 0.517637 | 2.532502 | 0.496648 |  |
| C | 3.196993 | 2.513944 | 0.470655 |  |
| C | 4.512384 | 2.49122 | 0.027192 |  |
| C | 2.560027 | 3.741133 | 0.762524 |  |
| C | 6.36721 | -1.13934 | -0.57303 |  |
| C | 6.235266 | 1.252995 | -0.94938 |  |
| C | 6.932889 | 0.067595 | -0.96461 |  |
| C | 1.19751 | 3.780924 | 0.728298 |  |
| C | -2.91667 | 3.739733 | -0.06446 |  |
| C | -1.58802 | 3.776225 | 0.248965 |  |
| C | -0.84756 | 2.549346 | 0.216256 |  |
| C | -0.89593 | 5.002788 | 0.575297 |  |
| C | 0.435628 | 5.004261 | 0.804426 |  |
| C | 4.546956 | -2.52939 | -0.10772 |  |
| C | 3.169998 | -2.60701 | -0.09211 |  |
| C | 2.5137 | -3.83834 | 0.144687 |  |
| C | 1.160043 | -3.85375 | 0.276408 |  |
| H | 6.750145 | 2.192654 | -1.13068 |  |
| H | 5.185837 | 3.327884 | 0.164791 |  |
| H | 3.15819 | 4.638104 | 0.858374 |  |
| H | 0.970675 | 5.920615 | 1.025071 |  |
| H | 6.958871 | -2.05049 | -0.63182 |  |
| H | 7.971447 | 0.077465 | -1.26947 |  |
| C | -5.70623 | -1.18394 | -0.23093 |  |
| C | -5.03077 | -2.37968 | 0.073772 |  |
| C | -7.10123 | -1.16028 | -0.46052 |  |
| C | -3.66246 | -2.42133 | 0.267887 |  |
| C | -3.0228 | -3.66805 | 0.526152 |  |
| C | -1.65902 | -3.75483 | 0.51963 |  |
| C | -0.94716 | -5.00749 | 0.654012 |  |
| C | 0.399377 | -5.0497 | 0.552808 |  |
| C | -7.04297 | 1.224704 | -0.80621 |  |
| C | -7.75293 | 0.029754 | -0.75544 |  |
| H | -7.65194 | -2.09198 | -0.39945 |  |
| H | -5.59542 | -3.30446 | 0.125063 |  |
| H | -3.63513 | -4.55205 | 0.662911 |  |
| H | -1.52727 | -5.90517 | 0.833826 |  |
| H | 0.943831 | -5.98125 | 0.655733 |  |
| H | 3.109799 | -4.7337 | 0.267067 |  |
| H | 5.179403 | -3.40638 | -0.16874 |  |
| H | -1.47472 | 5.918396 | 0.613202 |  |
| H | -3.50203 | 4.651311 | -0.10428 |  |
| H | -5.47279 | 3.396796 | -0.60192 |  |
| H | -7.55119 | 2.160377 | -1.00944 |  |
| H | -8.82228 | 0.029889 | -0.93409 |  |
| N | -1.42511 | 1.330288 | -0.109 |  |
| N | 1.121886 | 1.298351 | 0.591685 |  |
| N | 2.559777 | 1.278934 | 0.52478 |  |
| N | 4.922593 | 1.343175 | -0.56646 |  |
| N | -1.48668 | -1.29639 | 0.336628 |  |
| N | 1.0693 | -1.42563 | -0.25567 |  |
| N | 2.484191 | -1.42033 | -0.38647 |  |
| N | 5.073971 | -1.26997 | -0.19869 |  |
| Cu | -0.11827 | -0.0172 | 0.085376 |  |
| Cu | 3.683311 | -0.00993 | -0.24416 |  |

Cu-N4/Gr

| Symbol | X | Y | Z |  |
| --- | --- | --- | --- | --- |
| C | 5.551964 | -1.222619 | -0.000002 | 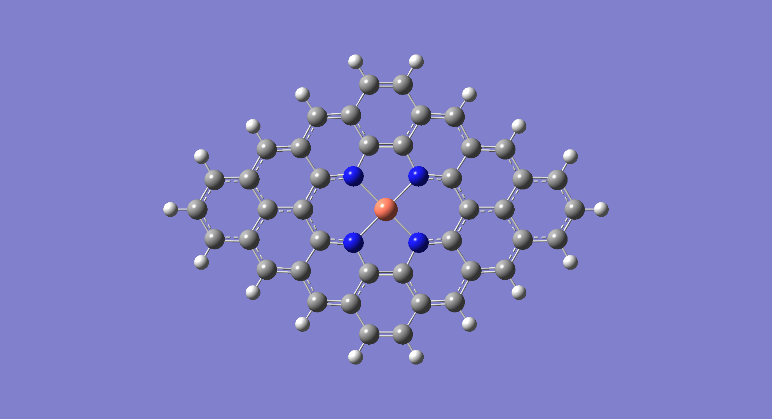 |
| C | 4.810101 | -0.000041 | 0 |  |
| C | 3.368147 | -0.00002 | 0.000003 |  |
| C | 2.672051 | -1.26399 | 0.000003 |  |
| C | 3.464126 | -2.493163 | -0.000003 |  |
| C | 4.842733 | -2.444692 | -0.000004 |  |
| C | 2.672117 | 1.264083 | 0.000004 |  |
| C | 0.689948 | 2.59516 | 0 |  |
| C | -0.689863 | 2.595152 | -0.000001 |  |
| C | -2.672108 | -1.26407 | 0.000003 |  |
| C | -0.689968 | -2.595206 | 0 |  |
| C | -3.368172 | 0.000051 | 0.000003 |  |
| C | -3.464283 | -2.493228 | -0.000002 |  |
| C | -4.810095 | 0.000037 | 0 |  |
| C | -4.84286 | -2.44468 | -0.000003 |  |
| C | -2.790782 | -3.757764 | -0.000006 |  |
| C | -5.551977 | 1.222628 | -0.000002 |  |
| C | -5.55204 | -1.222528 | -0.000001 |  |
| C | -6.964255 | 1.201488 | -0.000003 |  |
| C | -6.96425 | -1.201285 | -0.000003 |  |
| C | -7.664518 | 0.00014 | -0.000003 |  |
| C | -1.424171 | -3.825001 | -0.000006 |  |
| C | 2.790673 | -3.757713 | -0.000007 |  |
| C | 1.424069 | -3.824944 | -0.000007 |  |
| C | 0.689875 | -2.595111 | 0 |  |
| C | 0.676875 | -5.066644 | -0.000013 |  |
| C | -0.67695 | -5.066688 | -0.000012 |  |
| C | -2.672098 | 1.264031 | 0.000002 |  |
| C | -4.842754 | 2.444697 | -0.000004 |  |
| C | -3.464135 | 2.493183 | -0.000003 |  |
| C | -2.790671 | 3.757723 | -0.000008 |  |
| C | -1.424063 | 3.824949 | -0.000007 |  |
| H | -7.496346 | -2.146262 | -0.000004 |  |
| H | -5.402909 | -3.374317 | -0.000006 |  |
| H | -3.389628 | -4.661806 | -0.00001 |  |
| H | -1.235306 | -5.996132 | -0.000017 |  |
| H | -7.496265 | 2.146512 | -0.000005 |  |
| H | -8.74769 | 0.000126 | -0.000004 |  |
| C | 5.552013 | 1.222526 | -0.000001 |  |
| C | 4.842843 | 2.444695 | -0.000003 |  |
| C | 6.964224 | 1.201306 | -0.000003 |  |
| C | 3.464284 | 2.493267 | -0.000002 |  |
| C | 2.79078 | 3.757778 | -0.000006 |  |
| C | 1.424171 | 3.824966 | -0.000006 |  |
| C | 0.676967 | 5.066654 | -0.000012 |  |
| C | -0.676858 | 5.066652 | -0.000013 |  |
| C | 6.964242 | -1.201476 | -0.000004 |  |
| C | 7.664495 | -0.000118 | -0.000004 |  |
| H | 7.496314 | 2.146287 | -0.000004 |  |
| H | 5.40292 | 3.374315 | -0.000006 |  |
| H | 3.38961 | 4.661835 | -0.00001 |  |
| H | 1.235351 | 5.996085 | -0.000016 |  |
| H | -1.235246 | 5.996078 | -0.000017 |  |
| H | -3.389514 | 4.661763 | -0.000012 |  |
| H | -5.402725 | 3.374384 | -0.000007 |  |
| H | 1.235295 | -5.996054 | -0.000017 |  |
| H | 3.389546 | -4.661738 | -0.000012 |  |
| H | 5.402697 | -3.37438 | -0.000007 |  |
| H | 7.496258 | -2.146496 | -0.000005 |  |
| H | 8.747666 | -0.000095 | -0.000005 |  |
| N | 1.319748 | -1.345658 | 0.000006 |  |
| N | -1.319942 | -1.345808 | 0.000007 |  |
| N | 1.319943 | 1.345713 | 0.000007 |  |
| N | -1.319803 | 1.345653 | 0.000006 |  |
| Cu | 0.000047 | -0.000019 | 0.000027 |  |

Figure 2 (Cu2-N6/Gr (02))

| Sub Figure | Symbol | X | Y | Z |
| --- | --- | --- | --- | --- |
| (a) | C | -5.73027 | -1.16579 | -0.80206 |
|  | C | -5.02315 | 0.021706 | -0.42114 |
|  | C | -3.65754 | -0.04228 | -0.1124 |
|  | C | -2.9485 | -1.25276 | -0.22687 |
|  | C | -3.65202 | -2.44407 | -0.56301 |
|  | C | -5.01929 | -2.38848 | -0.8549 |
|  | C | 0.817558 | 2.368535 | -0.02508 |
|  | C | 3.579104 | 0.062449 | -0.20889 |
|  | C | 3.460671 | -2.27969 | 0.173269 |
|  | C | 4.954112 | 0.012381 | -0.46846 |
|  | C | 4.827855 | -2.39374 | -0.05413 |
|  | C | 2.622383 | -3.43183 | 0.298932 |
|  | C | 5.664893 | 1.210845 | -0.82769 |
|  | C | 5.62819 | -1.25556 | -0.37068 |
|  | C | 7.063156 | 1.088101 | -1.0767 |
|  | C | 7.005448 | -1.30854 | -0.62704 |
|  | C | 7.701278 | -0.13676 | -0.97208 |
|  | C | 1.247377 | -3.44703 | 0.220312 |
|  | C | -2.88049 | -3.63929 | -0.55336 |
|  | C | -1.53739 | -3.60507 | -0.26975 |
|  | C | -0.8651 | -2.33845 | -0.01907 |
|  | C | -0.7428 | -4.80464 | -0.16153 |
|  | C | 0.577337 | -4.72514 | 0.09575 |
|  | C | 2.871516 | 1.28143 | -0.30847 |
|  | C | 4.95737 | 2.426492 | -0.90233 |
|  | C | 3.577376 | 2.474484 | -0.64371 |
|  | C | 2.802808 | 3.668177 | -0.62172 |
|  | C | 1.4647 | 3.636918 | -0.29966 |
|  | H | 7.528366 | -2.25532 | -0.56053 |
|  | H | 5.280047 | -3.37787 | -0.04961 |
|  | H | 3.12225 | -4.39109 | 0.342464 |
|  | H | 1.18958 | -5.61369 | 0.178776 |
|  | H | 7.622707 | 1.973585 | -1.35629 |
|  | H | 8.766177 | -0.1975 | -1.1668 |
|  | C | -5.68679 | 1.28502 | -0.30353 |
|  | C | -4.88318 | 2.40871 | 0.034807 |
|  | C | -7.06682 | 1.339986 | -0.57418 |
|  | C | -3.51775 | 2.288055 | 0.294035 |
|  | C | -2.6727 | 3.427565 | 0.43724 |
|  | C | -1.29726 | 3.434784 | 0.313313 |
|  | C | -0.64839 | 4.724139 | 0.134551 |
|  | C | 0.658621 | 4.826252 | -0.17933 |
|  | C | -7.1207 | -1.04888 | -1.06148 |
|  | C | -7.7588 | 0.181077 | -0.94993 |
|  | H | -7.59017 | 2.285404 | -0.49192 |
|  | H | -5.33438 | 3.39208 | 0.084083 |
|  | H | -3.1678 | 4.389877 | 0.463821 |
|  | H | -1.27291 | 5.602887 | 0.229906 |
|  | H | 1.127801 | 5.790376 | -0.33891 |
|  | H | 3.281882 | 4.619379 | -0.82948 |
|  | H | 5.489047 | 3.341191 | -1.13846 |
|  | H | -1.23771 | -5.76083 | -0.28436 |
|  | H | -3.36385 | -4.5912 | -0.74476 |
|  | H | -5.54342 | -3.29832 | -1.12497 |
|  | H | -7.6836 | -1.93499 | -1.33147 |
|  | H | -8.82216 | 0.245596 | -1.15075 |
|  | N | -0.51671 | 2.286235 | 0.273834 |
|  | N | -2.93936 | 1.029106 | 0.349215 |
|  | N | -1.5908 | -1.16962 | -0.01047 |
|  | N | 0.462523 | -2.28252 | 0.20168 |
|  | N | 2.860747 | -1.04548 | 0.146789 |
|  | N | 1.529268 | 1.230441 | -0.02842 |
|  | Cu | -1.19181 | 0.583374 | 0.629525 |
|  | Cu | 1.207489 | -0.68173 | 0.806577 |
|  | O | -0.11428 | -0.21891 | 1.969953 |
|  | O | 1.223854 | 0.161526 | 2.509503 |
| (b) | C | -5.80022 | -1.15546 | -0.74182 |
|  | C | -5.06617 | 0.04377 | -0.45258 |
|  | C | -3.69266 | -0.01308 | -0.20584 |
|  | C | -2.99322 | -1.25454 | -0.24703 |
|  | C | -3.71822 | -2.44637 | -0.5137 |
|  | C | -5.10345 | -2.38432 | -0.76094 |
|  | C | 0.840341 | 2.400363 | 0.050185 |
|  | C | 3.576586 | 0.041242 | -0.26833 |
|  | C | 3.419519 | -2.29583 | -0.02258 |
|  | C | 4.946635 | -0.00657 | -0.56213 |
|  | C | 4.795 | -2.41945 | -0.28905 |
|  | C | 2.564618 | -3.4257 | 0.159644 |
|  | C | 5.674675 | 1.198622 | -0.83779 |
|  | C | 5.589279 | -1.28529 | -0.56074 |
|  | C | 7.061124 | 1.083439 | -1.11986 |
|  | C | 6.981179 | -1.33687 | -0.84752 |
|  | C | 7.685664 | -0.16577 | -1.11946 |
|  | C | 1.18293 | -3.42266 | 0.185115 |
|  | C | -2.95903 | -3.647 | -0.46476 |
|  | C | -1.60932 | -3.61303 | -0.19844 |
|  | C | -0.92516 | -2.34529 | 0.009519 |
|  | C | -0.81304 | -4.80903 | -0.08931 |
|  | C | 0.51694 | -4.71159 | 0.107548 |
|  | C | 2.875931 | 1.289243 | -0.24355 |
|  | C | 4.981934 | 2.426527 | -0.80348 |
|  | C | 3.600308 | 2.486744 | -0.5103 |
|  | C | 2.860595 | 3.691202 | -0.4294 |
|  | C | 1.508194 | 3.664306 | -0.14921 |
|  | H | 7.486294 | -2.29557 | -0.85032 |
|  | H | 5.242057 | -3.4058 | -0.30384 |
|  | H | 3.048654 | -4.39456 | 0.165617 |
|  | H | 1.139507 | -5.59455 | 0.176629 |
|  | H | 7.632944 | 1.97889 | -1.33458 |
|  | H | 8.74625 | -0.22497 | -1.33544 |
|  | C | -5.70745 | 1.324384 | -0.39067 |
|  | C | -4.90136 | 2.444839 | -0.11303 |
|  | C | -7.10808 | 1.382637 | -0.63292 |
|  | C | -3.51106 | 2.319377 | 0.109246 |
|  | C | -2.67016 | 3.455975 | 0.269434 |
|  | C | -1.28414 | 3.466001 | 0.249411 |
|  | C | -0.6214 | 4.755836 | 0.170336 |
|  | C | 0.708685 | 4.856916 | -0.03461 |
|  | C | -7.19359 | -1.03123 | -0.97545 |
|  | C | -7.81779 | 0.218567 | -0.91841 |
|  | H | -7.61404 | 2.340031 | -0.59321 |
|  | H | -5.34178 | 3.434035 | -0.09398 |
|  | H | -3.16443 | 4.419027 | 0.291937 |
|  | H | -1.24524 | 5.636984 | 0.247796 |
|  | H | 1.195714 | 5.821362 | -0.12304 |
|  | H | 3.359749 | 4.642205 | -0.58248 |
|  | H | 5.521043 | 3.348047 | -0.99398 |
|  | H | -1.30498 | -5.77092 | -0.1768 |
|  | H | -3.4517 | -4.60093 | -0.62132 |
|  | H | -5.64776 | -3.30145 | -0.95727 |
|  | H | -7.77379 | -1.91927 | -1.19818 |
|  | H | -8.8845 | 0.282138 | -1.10049 |
|  | N | -0.50699 | 2.313285 | 0.244624 |
|  | N | -2.94128 | 1.074045 | 0.103433 |
|  | N | -1.65633 | -1.1894 | -0.01147 |
|  | N | 0.403006 | -2.25997 | 0.224346 |
|  | N | 2.848132 | -1.05744 | 0.00088 |
|  | N | 1.547023 | 1.238225 | 0.039555 |
|  | Cu | -1.21066 | 0.595163 | 0.61862 |
|  | Cu | 1.243711 | -0.65567 | 0.94082 |
|  | O | -0.30972 | -0.15062 | 2.032531 |
|  | O | 2.151054 | -0.3982 | 2.511734 |
|  | H | 0.26404 | 0.23383 | 2.75035 |
| (c) | C | -5.72365 | -1.18268 | -0.91297 |
|  | C | -5.03242 | 0.010397 | -0.51793 |
|  | C | -3.6748 | -0.04988 | -0.17347 |
|  | C | -2.96244 | -1.25548 | -0.26336 |
|  | C | -3.65081 | -2.45307 | -0.59781 |
|  | C | -5.00839 | -2.40415 | -0.93252 |
|  | C | 0.755603 | 2.391684 | 0.064607 |
|  | C | 3.502412 | 0.090672 | -0.30962 |
|  | C | 3.434706 | -2.26502 | 0.068937 |
|  | C | 4.864339 | 0.048912 | -0.64592 |
|  | C | 4.779866 | -2.36649 | -0.24119 |
|  | C | 2.607478 | -3.41054 | 0.300196 |
|  | C | 5.55333 | 1.258156 | -1.01419 |
|  | C | 5.551285 | -1.21629 | -0.60769 |
|  | C | 6.937345 | 1.14766 | -1.34954 |
|  | C | 6.907108 | -1.25644 | -0.94303 |
|  | C | 7.581282 | -0.07352 | -1.30835 |
|  | C | 1.232043 | -3.42204 | 0.306373 |
|  | C | -2.87346 | -3.6433 | -0.53557 |
|  | C | -1.54119 | -3.59638 | -0.20736 |
|  | C | -0.87429 | -2.31831 | 0.033231 |
|  | C | -0.7543 | -4.79281 | -0.03326 |
|  | C | 0.562197 | -4.70474 | 0.236054 |
|  | C | 2.794723 | 1.311947 | -0.33403 |
|  | C | 4.845429 | 2.474541 | -1.01209 |
|  | C | 3.483817 | 2.515272 | -0.66939 |
|  | C | 2.71572 | 3.71052 | -0.56189 |
|  | C | 1.391807 | 3.672402 | -0.18586 |
|  | H | 7.437916 | -2.20105 | -0.92212 |
|  | H | 5.244512 | -3.34457 | -0.2596 |
|  | H | 3.108336 | -4.36918 | 0.345454 |
|  | H | 1.174739 | -5.5893 | 0.353886 |
|  | H | 7.474102 | 2.042085 | -1.64489 |
|  | H | 8.632664 | -0.12926 | -1.56771 |
|  | C | -5.7051 | 1.269121 | -0.41556 |
|  | C | -4.91526 | 2.392936 | -0.0355 |
|  | C | -7.07414 | 1.317757 | -0.72821 |
|  | C | -3.56539 | 2.276072 | 0.281162 |
|  | C | -2.74185 | 3.421562 | 0.506347 |
|  | C | -1.36674 | 3.443884 | 0.430402 |
|  | C | -0.71559 | 4.740612 | 0.320341 |
|  | C | 0.589917 | 4.856593 | 0.004213 |
|  | C | -7.10708 | -1.07219 | -1.2159 |
|  | C | -7.75066 | 0.15468 | -1.12632 |
|  | H | -7.60532 | 2.259919 | -0.65934 |
|  | H | -5.37211 | 3.373802 | 0.008642 |
|  | H | -3.25028 | 4.376245 | 0.54847 |
|  | H | -1.34042 | 5.613725 | 0.457665 |
|  | H | 1.058275 | 5.826996 | -0.11535 |
|  | H | 3.189563 | 4.667225 | -0.75681 |
|  | H | 5.363669 | 3.394933 | -1.25664 |
|  | H | -1.25016 | -5.75193 | -0.12593 |
|  | H | -3.34588 | -4.60272 | -0.7175 |
|  | H | -5.52235 | -3.31765 | -1.21009 |
|  | H | -7.65778 | -1.96131 | -1.50064 |
|  | H | -8.80765 | 0.216481 | -1.35955 |
|  | N | -0.58177 | 2.298328 | 0.366398 |
|  | N | -2.97215 | 1.019138 | 0.329747 |
|  | N | -1.61197 | -1.15896 | -0.01513 |
|  | N | 0.440983 | -2.25209 | 0.299128 |
|  | N | 2.808261 | -1.03969 | 0.050111 |
|  | N | 1.468171 | 1.260809 | 0.01747 |
|  | Cu | -1.23957 | 0.573794 | 0.656215 |
|  | Cu | 1.319052 | -0.72774 | 1.013659 |
|  | O | -0.16344 | -0.16908 | 1.961147 |
|  | O | 2.230644 | -0.25704 | 2.485045 |
|  | H | 0.274709 | 0.341004 | 2.692854 |
|  | H | 3.173201 | 0.002849 | 2.319038 |
| (d) | C | 5.806376 | 1.148649 | -0.56533 |
|  | C | 5.05594 | -0.05116 | -0.31379 |
|  | C | 3.680749 | 0.017226 | -0.07298 |
|  | C | 3.000009 | 1.259362 | -0.10996 |
|  | C | 3.738432 | 2.450576 | -0.36103 |
|  | C | 5.12391 | 2.385029 | -0.57774 |
|  | C | -0.92714 | -2.35543 | 0.057244 |
|  | C | -3.68208 | -0.01461 | -0.06447 |
|  | C | -3.46028 | 2.328961 | 0.131452 |
|  | C | -5.05696 | 0.055203 | -0.30996 |
|  | C | -4.83819 | 2.474931 | -0.08285 |
|  | C | -2.58616 | 3.453063 | 0.196982 |
|  | C | -5.80772 | -1.14376 | -0.56869 |
|  | C | -5.68224 | 1.351769 | -0.29388 |
|  | C | -7.20563 | -0.99865 | -0.79 |
|  | C | -7.07007 | 1.425845 | -0.5218 |
|  | C | -7.80316 | 0.255672 | -0.76171 |
|  | C | -1.20247 | 3.439604 | 0.156924 |
|  | C | 2.972253 | 3.649185 | -0.35938 |
|  | C | 1.610197 | 3.618777 | -0.1605 |
|  | C | 0.924828 | 2.357221 | 0.044989 |
|  | C | 0.803691 | 4.813224 | -0.13325 |
|  | C | -0.53129 | 4.720988 | 0.027319 |
|  | C | -3.00699 | -1.25733 | -0.10023 |
|  | C | -5.12637 | -2.38097 | -0.5877 |
|  | C | -3.74201 | -2.44718 | -0.36582 |
|  | C | -2.97177 | -3.64365 | -0.37484 |
|  | C | -1.60965 | -3.61407 | -0.17142 |
|  | H | -7.56355 | 2.390493 | -0.51496 |
|  | H | -5.25828 | 3.472547 | -0.11971 |
|  | H | -3.05959 | 4.426838 | 0.171799 |
|  | H | -1.15492 | 5.605478 | 0.044508 |
|  | H | -7.80148 | -1.8834 | -0.98295 |
|  | H | -8.87107 | 0.333417 | -0.9343 |
|  | C | 5.681825 | -1.34672 | -0.29839 |
|  | C | 4.840762 | -2.47097 | -0.08951 |
|  | C | 7.071686 | -1.41901 | -0.52353 |
|  | C | 3.461723 | -2.32638 | 0.125063 |
|  | C | 2.587993 | -3.45192 | 0.195901 |
|  | C | 1.204456 | -3.43992 | 0.16224 |
|  | C | 0.532255 | -4.71927 | 0.015893 |
|  | C | -0.80192 | -4.8086 | -0.15384 |
|  | C | 7.204642 | 1.006118 | -0.78316 |
|  | C | 7.804096 | -0.24833 | -0.75803 |
|  | H | 7.565728 | -2.38338 | -0.51743 |
|  | H | 5.262723 | -3.46788 | -0.12373 |
|  | H | 3.062215 | -4.42504 | 0.161972 |
|  | H | 1.155324 | -5.60433 | 0.02338 |
|  | H | -1.29266 | -5.76629 | -0.28406 |
|  | H | -3.4647 | -4.59528 | -0.54484 |
|  | H | -5.67956 | -3.29271 | -0.78185 |
|  | H | 1.29536 | 5.772478 | -0.2471 |
|  | H | 3.468345 | 4.601459 | -0.51551 |
|  | H | 5.677805 | 3.298708 | -0.76074 |
|  | H | 7.800444 | 1.892171 | -0.97014 |
|  | H | 8.872497 | -0.32415 | -0.92792 |
|  | N | 0.428295 | -2.27773 | 0.218703 |
|  | N | 2.912178 | -1.07319 | 0.200912 |
|  | N | 1.643133 | 1.204991 | 0.080292 |
|  | N | -0.42783 | 2.277022 | 0.192053 |
|  | N | -2.90943 | 1.075044 | 0.210496 |
|  | N | -1.64934 | -1.20515 | 0.106431 |
|  | Cu | 1.177116 | -0.59485 | 0.638853 |
|  | Cu | -1.1695 | 0.596789 | 0.635374 |
|  | O | 0.024849 | 0.01875 | 2.011718 |
|  | H | -0.37556 | -0.70498 | 2.553612 |
| (e) | C | 5.871831 | 1.087396 | -0.04376 |
|  | C | 5.074888 | -0.1088 | 0.018868 |
|  | C | 3.676108 | -0.02213 | -0.01989 |
|  | C | 3.052704 | 1.219785 | -0.17 |
|  | C | 3.819434 | 2.414202 | -0.2008 |
|  | C | 5.216735 | 2.338321 | -0.14453 |
|  | C | -0.97798 | -2.34897 | -0.13989 |
|  | C | -3.66866 | 0.014685 | 0.0037 |
|  | C | -3.38562 | 2.341117 | 0.101509 |
|  | C | -5.06852 | 0.104816 | 0.005925 |
|  | C | -4.76468 | 2.516589 | 0.128365 |
|  | C | -2.50113 | 3.457507 | 0.011652 |
|  | C | -5.86731 | -1.08957 | -0.08898 |
|  | C | -5.66681 | 1.408416 | 0.090043 |
|  | C | -7.28311 | -0.92312 | -0.07762 |
|  | C | -7.06401 | 1.505092 | 0.097987 |
|  | C | -7.84617 | 0.338451 | 0.018503 |
|  | C | -1.14205 | 3.411379 | -0.15409 |
|  | C | 3.062309 | 3.617633 | -0.2975 |
|  | C | 1.687565 | 3.589902 | -0.30916 |
|  | C | 0.97271 | 2.326436 | -0.26797 |
|  | C | 0.884907 | 4.786233 | -0.342 |
|  | C | -0.45521 | 4.68735 | -0.26977 |
|  | C | -3.04576 | -1.23348 | -0.08573 |
|  | C | -5.21761 | -2.33935 | -0.19666 |
|  | C | -3.81636 | -2.42068 | -0.20378 |
|  | C | -3.06327 | -3.62603 | -0.28034 |
|  | C | -1.68687 | -3.60497 | -0.24396 |
|  | H | -7.53688 | 2.478168 | 0.159202 |
|  | H | -5.16111 | 3.524539 | 0.112853 |
|  | H | -2.95932 | 4.438301 | 0.038305 |
|  | H | -1.08061 | 5.570811 | -0.27687 |
|  | H | -7.91399 | -1.80109 | -0.15734 |
|  | H | -8.92685 | 0.43398 | 0.023287 |
|  | C | 5.672069 | -1.40843 | 0.133102 |
|  | C | 4.775761 | -2.51925 | 0.144375 |
|  | C | 7.070518 | -1.4966 | 0.195546 |
|  | C | 3.395557 | -2.35532 | 0.095054 |
|  | C | 2.514468 | -3.47506 | 0.028677 |
|  | C | 1.149402 | -3.43363 | -0.09279 |
|  | C | 0.457384 | -4.70849 | -0.20606 |
|  | C | -0.88279 | -4.80112 | -0.29505 |
|  | C | 7.283959 | 0.931773 | 0.025456 |
|  | C | 7.849975 | -0.32937 | 0.140129 |
|  | H | 7.54467 | -2.46719 | 0.282084 |
|  | H | 5.176004 | -3.52429 | 0.19877 |
|  | H | 2.979015 | -4.45297 | 0.028305 |
|  | H | 1.079897 | -5.59388 | -0.22003 |
|  | H | -1.38128 | -5.75862 | -0.3905 |
|  | H | -3.58016 | -4.57647 | -0.36331 |
|  | H | -5.8071 | -3.24654 | -0.26161 |
|  | H | 1.383043 | 5.745953 | -0.4116 |
|  | H | 3.576688 | 4.572043 | -0.33956 |
|  | H | 5.804954 | 3.247835 | -0.18717 |
|  | H | 7.912214 | 1.814649 | -0.00415 |
|  | H | 8.929768 | -0.41958 | 0.191019 |
|  | N | 0.386757 | -2.25096 | -0.11535 |
|  | N | 2.824562 | -1.09445 | 0.097212 |
|  | N | 1.675725 | 1.169027 | -0.28261 |
|  | N | -0.38071 | 2.219456 | -0.20225 |
|  | N | -2.8218 | 1.087722 | 0.06518 |
|  | N | -1.66476 | -1.19746 | -0.04248 |
|  | Cu | 1.06863 | -0.555 | -0.26321 |
|  | Cu | -1.03492 | 0.530073 | 0.254803 |
|  | O | -0.22356 | 0.238088 | 2.157884 |
|  | H | -0.29528 | -0.63314 | 2.628267 |
|  | H | 0.422572 | 0.816213 | 2.641285 |

Figure 3 (Cu2-N6/Gr (01))

| Sub Figure | Symbol | X | Y | Z |
| --- | --- | --- | --- | --- |
| (a) | C | -5.7841 | -1.16307 | -0.6642 |
|  | C | -5.04027 | 0.029536 | -0.35529 |
|  | C | -3.66877 | -0.05255 | -0.10861 |
|  | C | -3.00171 | -1.28505 | -0.19165 |
|  | C | -3.71466 | -2.46737 | -0.51558 |
|  | C | -5.1023 | -2.39818 | -0.73545 |
|  | C | 0.93138 | 2.336363 | -0.11487 |
|  | C | 3.710433 | 0.017122 | -0.08103 |
|  | C | 3.495068 | -2.31613 | 0.217129 |
|  | C | 5.082939 | -0.06565 | -0.33783 |
|  | C | 4.86665 | -2.46579 | 0.000449 |
|  | C | 2.60772 | -3.43084 | 0.282646 |
|  | C | 5.824956 | 1.121081 | -0.64757 |
|  | C | 5.7104 | -1.34928 | -0.25114 |
|  | C | 7.218509 | 0.982954 | -0.85337 |
|  | C | 7.10104 | -1.42474 | -0.47076 |
|  | C | 7.828309 | -0.26765 | -0.76661 |
|  | C | 1.230417 | -3.40863 | 0.176121 |
|  | C | -2.92377 | -3.65221 | -0.53475 |
|  | C | -1.568 | -3.60957 | -0.28571 |
|  | C | -0.90016 | -2.3476 | -0.01643 |
|  | C | -0.74897 | -4.795 | -0.24873 |
|  | C | 0.572007 | -4.69279 | -0.00583 |
|  | C | 3.020729 | 1.242756 | -0.20049 |
|  | C | 5.133152 | 2.360268 | -0.69847 |
|  | C | 3.755523 | 2.433437 | -0.47797 |
|  | C | 2.98882 | 3.634742 | -0.50997 |
|  | C | 1.629646 | 3.602094 | -0.31707 |
|  | H | 7.600715 | -2.38454 | -0.41118 |
|  | H | 5.289561 | -3.46293 | 0.014351 |
|  | H | 3.075242 | -4.4075 | 0.271375 |
|  | H | 1.200871 | -5.57255 | 0.034951 |
|  | H | 7.81192 | 1.864278 | -1.06829 |
|  | H | 8.897408 | -0.34505 | -0.92839 |
|  | C | -5.66065 | 1.326216 | -0.31075 |
|  | C | -4.80242 | 2.445162 | -0.08064 |
|  | C | -7.04077 | 1.412608 | -0.54587 |
|  | C | -3.4299 | 2.289392 | 0.119757 |
|  | C | -2.55075 | 3.415805 | 0.149667 |
|  | C | -1.17219 | 3.413024 | 0.043292 |
|  | C | -0.51072 | 4.698713 | -0.0878 |
|  | C | 0.821243 | 4.794139 | -0.27591 |
|  | C | -7.18262 | -1.0036 | -0.88941 |
|  | C | -7.7774 | 0.247193 | -0.82231 |
|  | H | -7.53337 | 2.377382 | -0.52008 |
|  | H | -5.21228 | 3.446513 | -0.13151 |
|  | H | -3.03062 | 4.387136 | 0.161538 |
|  | H | -1.13351 | 5.583263 | -0.04501 |
|  | H | 1.310116 | 5.755224 | -0.38548 |
|  | H | 3.487222 | 4.584478 | -0.6726 |
|  | H | 5.685341 | 3.265795 | -0.92379 |
|  | H | -1.22179 | -5.75679 | -0.41015 |
|  | H | -3.39607 | -4.60829 | -0.73495 |
|  | H | -5.65682 | -3.30422 | -0.9501 |
|  | H | -7.77791 | -1.87805 | -1.12656 |
|  | H | -8.84359 | 0.33349 | -1.00017 |
|  | N | -0.3926 | 2.244749 | 0.032287 |
|  | N | -2.88562 | 1.03327 | 0.166677 |
|  | N | -1.65247 | -1.23121 | 0.061854 |
|  | N | 0.450091 | -2.24921 | 0.176703 |
|  | N | 2.950953 | -1.04816 | 0.308853 |
|  | N | 1.661982 | 1.163243 | -0.07173 |
|  | Cu | -1.23122 | 0.472858 | 0.749266 |
|  | Cu | 1.195594 | -0.56169 | 0.528459 |
|  | O | -0.03408 | -0.23976 | 1.927404 |
|  | O | -0.73325 | 0.948267 | 2.513528 |
| (b) | C | 5.786866 | 1.166229 | -0.64284 |
|  | C | 5.037356 | -0.043 | -0.45099 |
|  | C | 3.665815 | 0.011065 | -0.17546 |
|  | C | 2.98366 | 1.252459 | -0.11084 |
|  | C | 3.723057 | 2.450597 | -0.32425 |
|  | C | 5.104116 | 2.39836 | -0.57175 |
|  | C | -0.90693 | -2.35383 | 0.085234 |
|  | C | -3.66239 | -0.0148 | -0.1744 |
|  | C | -3.45535 | 2.338197 | -0.09057 |
|  | C | -5.03292 | 0.041536 | -0.45836 |
|  | C | -4.82045 | 2.470754 | -0.36051 |
|  | C | -2.59232 | 3.468923 | 0.012247 |
|  | C | -5.78239 | -1.16886 | -0.65478 |
|  | C | -5.65774 | 1.335435 | -0.54006 |
|  | C | -7.17363 | -1.03928 | -0.92062 |
|  | C | -7.0375 | 1.396248 | -0.80829 |
|  | C | -7.76816 | 0.213886 | -0.99094 |
|  | C | -1.21389 | 3.456431 | 0.077734 |
|  | C | 2.967052 | 3.652765 | -0.27603 |
|  | C | 1.605193 | 3.621477 | -0.09796 |
|  | C | 0.912358 | 2.35717 | 0.071759 |
|  | C | 0.807302 | 4.822064 | -0.07585 |
|  | C | -0.53358 | 4.736898 | 0.010063 |
|  | C | -2.9847 | -1.2545 | -0.1073 |
|  | C | -5.10059 | -2.40266 | -0.578 |
|  | C | -3.72313 | -2.45419 | -0.32157 |
|  | C | -2.96321 | -3.65319 | -0.266 |
|  | C | -1.60102 | -3.61803 | -0.08483 |
|  | H | -7.52817 | 2.360054 | -0.87497 |
|  | H | -5.2416 | 3.464443 | -0.45035 |
|  | H | -3.06642 | 4.440384 | -0.05251 |
|  | H | -1.15427 | 5.623571 | 0.007688 |
|  | H | -7.76544 | -1.93516 | -1.06939 |
|  | H | -8.83105 | 0.281717 | -1.19589 |
|  | C | 5.662083 | -1.33587 | -0.53206 |
|  | C | 4.831517 | -2.47099 | -0.35963 |
|  | C | 7.047601 | -1.3957 | -0.7949 |
|  | C | 3.458287 | -2.34014 | -0.09733 |
|  | C | 2.598052 | -3.46985 | 0.001658 |
|  | C | 1.216795 | -3.45618 | 0.073733 |
|  | C | 0.537222 | -4.73668 | 0.013413 |
|  | C | -0.80459 | -4.81904 | -0.06433 |
|  | C | 7.179712 | 1.03861 | -0.90206 |
|  | C | 7.778179 | -0.21498 | -0.9712 |
|  | H | 7.537772 | -2.35985 | -0.86011 |
|  | H | 5.254761 | -3.4639 | -0.44837 |
|  | H | 3.070931 | -4.44183 | -0.06232 |
|  | H | 1.156519 | -5.62424 | 0.009688 |
|  | H | -1.31146 | -5.77477 | -0.13113 |
|  | H | -3.46154 | -4.60883 | -0.39148 |
|  | H | -5.64692 | -3.32608 | -0.73235 |
|  | H | 1.312049 | 5.778634 | -0.1459 |
|  | H | 3.469308 | 4.606257 | -0.40123 |
|  | H | 5.651066 | 3.322414 | -0.7205 |
|  | H | 7.771246 | 1.935131 | -1.04808 |
|  | H | 8.842006 | -0.28066 | -1.17069 |
|  | N | 0.451139 | -2.29039 | 0.162153 |
|  | N | 2.903932 | -1.09575 | 0.031478 |
|  | N | 1.624449 | 1.196929 | 0.115054 |
|  | N | -0.44354 | 2.289596 | 0.163278 |
|  | N | -2.89797 | 1.09128 | 0.045635 |
|  | N | -1.62424 | -1.19422 | 0.12471 |
|  | Cu | 1.218316 | -0.64733 | 0.672952 |
|  | Cu | -1.20473 | 0.637548 | 0.637524 |
|  | O | -0.52537 | 0.579172 | 2.466548 |
|  | O | 0.525112 | -0.56353 | 2.377722 |
|  | H | -1.07372 | 0.254907 | 3.230654 |
| (c) | C | -5.81105 | -1.15023 | -0.6141 |
|  | C | -5.06762 | 0.05941 | -0.3888 |
|  | C | -3.69997 | -0.01619 | -0.09626 |
|  | C | -3.03329 | -1.24388 | -0.07943 |
|  | C | -3.75547 | -2.44598 | -0.27439 |
|  | C | -5.13212 | -2.39027 | -0.54057 |
|  | C | 0.921115 | 2.301356 | 0.154607 |
|  | C | 3.699969 | 0.016156 | -0.09627 |
|  | C | 3.482531 | -2.33783 | 0.079305 |
|  | C | 5.067633 | -0.05944 | -0.38878 |
|  | C | 4.824667 | -2.48865 | -0.22214 |
|  | C | 2.582651 | -3.44825 | 0.212667 |
|  | C | 5.811057 | 1.150197 | -0.61407 |
|  | C | 5.683844 | -1.35488 | -0.43461 |
|  | C | 7.203619 | 1.01403 | -0.88654 |
|  | C | 7.053328 | -1.421 | -0.70735 |
|  | C | 7.788856 | -0.23882 | -0.92895 |
|  | C | 1.210713 | -3.41933 | 0.258654 |
|  | C | -2.97425 | -3.63193 | -0.18811 |
|  | C | -1.61442 | -3.57873 | 0.019762 |
|  | C | -0.92116 | -2.30141 | 0.154821 |
|  | C | -0.81832 | -4.77736 | 0.1244 |
|  | C | 0.518411 | -4.69503 | 0.246882 |
|  | C | 3.033265 | 1.243819 | -0.07964 |
|  | C | 5.132108 | 2.39024 | -0.54056 |
|  | C | 3.755451 | 2.445941 | -0.27448 |
|  | C | 2.974233 | 3.631886 | -0.18816 |
|  | C | 1.614392 | 3.578674 | 0.019689 |
|  | H | 7.548679 | -2.38392 | -0.74894 |
|  | H | 5.235949 | -3.48697 | -0.30282 |
|  | H | 3.040194 | -4.42971 | 0.185748 |
|  | H | 1.131769 | -5.58406 | 0.313789 |
|  | H | 7.797315 | 1.905374 | -1.05313 |
|  | H | 8.850401 | -0.3188 | -1.13644 |
|  | C | -5.68385 | 1.354852 | -0.43463 |
|  | C | -4.82468 | 2.488625 | -0.22206 |
|  | C | -7.05331 | 1.420991 | -0.70741 |
|  | C | -3.48253 | 2.337787 | 0.079337 |
|  | C | -2.58268 | 3.448222 | 0.212778 |
|  | C | -1.21074 | 3.419312 | 0.258641 |
|  | C | -0.51842 | 4.694985 | 0.246919 |
|  | C | 0.818316 | 4.777299 | 0.124427 |
|  | C | -7.20361 | -1.01404 | -0.88661 |
|  | C | -7.78884 | 0.238803 | -0.92904 |
|  | H | -7.54866 | 2.383902 | -0.749 |
|  | H | -5.23599 | 3.486937 | -0.30264 |
|  | H | -3.04024 | 4.429681 | 0.18603 |
|  | H | -1.13175 | 5.584031 | 0.313894 |
|  | H | 1.325704 | 5.734332 | 0.093818 |
|  | H | 3.455219 | 4.599246 | -0.289 |
|  | H | 5.681286 | 3.309376 | -0.70993 |
|  | H | -1.32571 | -5.73439 | 0.093755 |
|  | H | -3.45523 | -4.59929 | -0.28907 |
|  | H | -5.68128 | -3.3094 | -0.71001 |
|  | H | -7.7973 | -1.90539 | -1.05321 |
|  | H | -8.85038 | 0.318787 | -1.13656 |
|  | N | -0.43586 | 2.234004 | 0.262207 |
|  | N | -2.93621 | 1.076691 | 0.229735 |
|  | N | -1.665 | -1.16012 | 0.111451 |
|  | N | 0.435822 | -2.23405 | 0.262482 |
|  | N | 2.936225 | -1.07673 | 0.229873 |
|  | N | 1.66494 | 1.160031 | 0.111072 |
|  | Cu | -1.23383 | 0.589407 | 0.627537 |
|  | Cu | 1.233823 | -0.58932 | 0.627489 |
|  | O | 0.000197 | 0.000389 | 1.703827 |
| (d) | C | -5.80813 | -1.14809 | -0.56049 |
|  | C | -5.05692 | 0.052254 | -0.31326 |
|  | C | -3.68141 | -0.01596 | -0.07301 |
|  | C | -3.0007 | -1.25903 | -0.10762 |
|  | C | -3.7409 | -2.45113 | -0.35428 |
|  | C | -5.12582 | -2.38533 | -0.56954 |
|  | C | 0.928782 | 2.35582 | 0.058584 |
|  | C | 3.682954 | 0.014513 | -0.06385 |
|  | C | 3.45983 | -2.32963 | 0.125684 |
|  | C | 5.057993 | -0.0554 | -0.30751 |
|  | C | 4.838191 | -2.47577 | -0.08786 |
|  | C | 2.58619 | -3.45368 | 0.186545 |
|  | C | 5.809725 | 1.143842 | -0.56298 |
|  | C | 5.6828 | -1.35234 | -0.2949 |
|  | C | 7.208003 | 0.998123 | -0.78363 |
|  | C | 7.070697 | -1.42687 | -0.52219 |
|  | C | 7.804923 | -0.25641 | -0.75806 |
|  | C | 1.201763 | -3.44003 | 0.148457 |
|  | C | -2.97481 | -3.64972 | -0.35151 |
|  | C | -1.61187 | -3.61898 | -0.15687 |
|  | C | -0.92615 | -2.3576 | 0.044056 |
|  | C | -0.80557 | -4.81352 | -0.13195 |
|  | C | 0.530244 | -4.72135 | 0.022119 |
|  | C | 3.008605 | 1.257558 | -0.0977 |
|  | C | 5.129208 | 2.381049 | -0.58134 |
|  | C | 3.743964 | 2.447142 | -0.36183 |
|  | C | 2.974143 | 3.643741 | -0.37102 |
|  | C | 1.611882 | 3.614556 | -0.16924 |
|  | H | 7.563419 | -2.39193 | -0.5181 |
|  | H | 5.257456 | -3.47355 | -0.12992 |
|  | H | 3.05936 | -4.42753 | 0.159796 |
|  | H | 1.153892 | -5.60587 | 0.037195 |
|  | H | 7.804281 | 1.882921 | -0.97512 |
|  | H | 8.872864 | -0.33436 | -0.93026 |
|  | C | -5.68209 | 1.348231 | -0.30135 |
|  | C | -4.84002 | 2.472936 | -0.09619 |
|  | C | -7.07189 | 1.420381 | -0.52578 |
|  | C | -3.46193 | 2.328278 | 0.119124 |
|  | C | -2.58641 | 3.453395 | 0.188977 |
|  | C | -1.20321 | 3.441021 | 0.158438 |
|  | C | -0.53023 | 4.720356 | 0.013011 |
|  | C | 0.804253 | 4.8094 | -0.15365 |
|  | C | -7.20586 | -1.00573 | -0.77775 |
|  | C | -7.8046 | 0.249358 | -0.75614 |
|  | H | -7.56556 | 2.384938 | -0.52234 |
|  | H | -5.26164 | 3.469864 | -0.13358 |
|  | H | -3.06017 | 4.426681 | 0.152513 |
|  | H | -1.15316 | 5.605553 | 0.018762 |
|  | H | 1.295472 | 5.766936 | -0.28322 |
|  | H | 3.467525 | 4.595278 | -0.54038 |
|  | H | 5.682978 | 3.293031 | -0.77267 |
|  | H | -1.29788 | -5.7728 | -0.24276 |
|  | H | -3.47134 | -4.60227 | -0.50444 |
|  | H | -5.68054 | -3.29905 | -0.7498 |
|  | H | -7.80234 | -1.89198 | -0.96154 |
|  | H | -8.87312 | 0.325015 | -0.92561 |
|  | N | -0.42702 | 2.278663 | 0.217703 |
|  | N | -2.91305 | 1.074527 | 0.198097 |
|  | N | -1.6442 | -1.20496 | 0.078983 |
|  | N | 0.428364 | -2.27687 | 0.185443 |
|  | N | 2.909133 | -1.07587 | 0.206395 |
|  | N | 1.650942 | 1.205918 | 0.109695 |
|  | Cu | -1.179 | 0.597368 | 0.639792 |
|  | Cu | 1.169906 | -0.60023 | 0.635768 |
|  | O | -0.02792 | -0.02281 | 2.006936 |
|  | H | 0.372494 | 0.692435 | 2.55952 |
| (e) | C | 5.87183 | 1.087489 | -0.04459 |
|  | C | 5.074925 | -0.1087 | 0.018646 |
|  | C | 3.676145 | -0.02204 | -0.01974 |
|  | C | 3.052665 | 1.219835 | -0.17017 |
|  | C | 3.819373 | 2.414245 | -0.20152 |
|  | C | 5.216697 | 2.338381 | -0.14558 |
|  | C | -0.97794 | -2.34891 | -0.14009 |
|  | C | -3.66867 | 0.014686 | 0.003783 |
|  | C | -3.38569 | 2.341124 | 0.102096 |
|  | C | -5.06854 | 0.104812 | 0.005681 |
|  | C | -4.76476 | 2.516585 | 0.128622 |
|  | C | -2.50117 | 3.457505 | 0.012398 |
|  | C | -5.8673 | -1.08956 | -0.0897 |
|  | C | -5.66686 | 1.408395 | 0.089935 |
|  | C | -7.2831 | -0.92313 | -0.07862 |
|  | C | -7.06406 | 1.505046 | 0.09758 |
|  | C | -7.8462 | 0.338417 | 0.017661 |
|  | C | -1.1421 | 3.411381 | -0.1535 |
|  | C | 3.062205 | 3.617645 | -0.29827 |
|  | C | 1.687452 | 3.589905 | -0.3095 |
|  | C | 0.972606 | 2.326445 | -0.2679 |
|  | C | 0.884786 | 4.786233 | -0.34212 |
|  | C | -0.4553 | 4.687348 | -0.26938 |
|  | C | -3.04572 | -1.23343 | -0.08591 |
|  | C | -5.21755 | -2.3393 | -0.19751 |
|  | C | -3.8163 | -2.42061 | -0.20438 |
|  | C | -3.06321 | -3.62596 | -0.28107 |
|  | C | -1.68681 | -3.6049 | -0.24446 |
|  | H | -7.53695 | 2.478106 | 0.15889 |
|  | H | -5.1612 | 3.52453 | 0.113126 |
|  | H | -2.95935 | 4.438303 | 0.039128 |
|  | H | -1.0807 | 5.570813 | -0.27626 |
|  | H | -7.91395 | -1.80108 | -0.15867 |
|  | H | -8.92687 | 0.433932 | 0.022223 |
|  | C | 5.67215 | -1.4083 | 0.133092 |
|  | C | 4.775848 | -2.51915 | 0.144785 |
|  | C | 7.070608 | -1.49642 | 0.195186 |
|  | C | 3.395634 | -2.35525 | 0.095778 |
|  | C | 2.514502 | -3.47501 | 0.029358 |
|  | C | 1.149439 | -3.43362 | -0.09259 |
|  | C | 0.45744 | -4.70846 | -0.20613 |
|  | C | -0.88272 | -4.80105 | -0.29551 |
|  | C | 7.283984 | 0.931902 | 0.024292 |
|  | C | 7.850043 | -0.3292 | 0.139209 |
|  | H | 7.544803 | -2.46699 | 0.281866 |
|  | H | 5.176121 | -3.52418 | 0.199117 |
|  | H | 2.979064 | -4.45291 | 0.029085 |
|  | H | 1.079936 | -5.59386 | -0.21999 |
|  | H | -1.38119 | -5.75854 | -0.39118 |
|  | H | -3.58009 | -4.57638 | -0.36428 |
|  | H | -5.80701 | -3.24648 | -0.26281 |
|  | H | 1.382892 | 5.745954 | -0.41191 |
|  | H | 3.576559 | 4.572054 | -0.34064 |
|  | H | 5.804897 | 3.247887 | -0.18863 |
|  | H | 7.912215 | 1.81478 | -0.00577 |
|  | H | 8.929849 | -0.41938 | 0.189834 |
|  | N | 0.386786 | -2.25096 | -0.11539 |
|  | N | 2.824665 | -1.09437 | 0.098052 |
|  | N | 1.675639 | 1.169049 | -0.28251 |
|  | N | -0.38076 | 2.219451 | -0.20178 |
|  | N | -2.82187 | 1.087735 | 0.065861 |
|  | N | -1.66469 | -1.19741 | -0.04254 |
|  | Cu | 1.068809 | -0.55502 | -0.26225 |
|  | Cu | -1.03506 | 0.530148 | 0.25531 |
|  | O | -0.22364 | 0.23686 | 2.158345 |
|  | H | -0.29645 | -0.63471 | 2.627935 |
|  | H | 0.423426 | 0.813643 | 2.642092 |
| (f) | C | 5.537225 | 1.194845 | -1.16565 |
|  | C | 4.839495 | -0.02262 | -0.88496 |
|  | C | 3.556108 | 0.023177 | -0.32037 |
|  | C | 2.901899 | 1.249477 | -0.07856 |
|  | C | 3.602648 | 2.462028 | -0.32899 |
|  | C | 4.896372 | 2.423956 | -0.86333 |
|  | C | -0.88908 | -2.32284 | 0.384067 |
|  | C | -3.57883 | -0.01778 | -0.31718 |
|  | C | -3.39497 | 2.3359 | -0.20398 |
|  | C | -4.87839 | 0.038056 | -0.83649 |
|  | C | -4.67282 | 2.47026 | -0.73239 |
|  | C | -2.58036 | 3.464766 | 0.138211 |
|  | C | -5.59422 | -1.17706 | -1.09795 |
|  | C | -5.4687 | 1.326233 | -1.05867 |
|  | C | -6.91896 | -1.05632 | -1.60583 |
|  | C | -6.77609 | 1.380399 | -1.56424 |
|  | C | -7.47813 | 0.192032 | -1.83212 |
|  | C | -1.23969 | 3.449464 | 0.436478 |
|  | C | 2.892042 | 3.659473 | -0.02697 |
|  | C | 1.566728 | 3.607482 | 0.326097 |
|  | C | 0.871078 | 2.329666 | 0.41973 |
|  | C | 0.78496 | 4.79833 | 0.564414 |
|  | C | -0.55802 | 4.717808 | 0.616606 |
|  | C | -2.93501 | -1.24504 | -0.08864 |
|  | C | -4.95311 | -2.40813 | -0.83035 |
|  | C | -3.6402 | -2.45114 | -0.33587 |
|  | C | -2.91352 | -3.64932 | -0.08222 |
|  | C | -1.58319 | -3.60123 | 0.250115 |
|  | H | -7.24491 | 2.341497 | -1.74031 |
|  | H | -5.08235 | 3.462419 | -0.87707 |
|  | H | -3.05468 | 4.43704 | 0.080573 |
|  | H | -1.17327 | 5.599228 | 0.744249 |
|  | H | -7.48925 | -1.95601 | -1.80721 |
|  | H | -8.48891 | 0.25705 | -2.21877 |
|  | C | 5.428125 | -1.30558 | -1.11696 |
|  | C | 4.651534 | -2.45427 | -0.77037 |
|  | C | 6.725516 | -1.34821 | -1.66153 |
|  | C | 3.387133 | -2.32861 | -0.20375 |
|  | C | 2.566729 | -3.45172 | 0.152185 |
|  | C | 1.221155 | -3.43375 | 0.436365 |
|  | C | 0.539628 | -4.7118 | 0.554684 |
|  | C | -0.8008 | -4.79557 | 0.462361 |
|  | C | 6.844407 | 1.08942 | -1.7076 |
|  | C | 7.407881 | -0.15865 | -1.95113 |
|  | H | 7.196071 | -2.30615 | -1.84988 |
|  | H | 5.067435 | -3.44307 | -0.92009 |
|  | H | 3.033161 | -4.42707 | 0.081952 |
|  | H | 1.155088 | -5.59548 | 0.664415 |
|  | H | -1.31521 | -5.74849 | 0.506412 |
|  | H | -3.4073 | -4.61 | -0.18384 |
|  | H | -5.47672 | -3.33639 | -1.02948 |
|  | H | 1.301702 | 5.746506 | 0.655577 |
|  | H | 3.388989 | 4.619314 | -0.11691 |
|  | H | 5.413516 | 3.354998 | -1.06748 |
|  | H | 7.404945 | 1.992855 | -1.91895 |
|  | H | 8.407785 | -0.215 | -2.36615 |
|  | N | 0.447492 | -2.25918 | 0.513216 |
|  | N | 2.867748 | -1.0811 | 0.058869 |
|  | N | 1.593359 | 1.149016 | 0.348099 |
|  | N | -0.46809 | 2.267895 | 0.48194 |
|  | N | -2.86341 | 1.089666 | 0.031726 |
|  | N | -1.61306 | -1.15782 | 0.31163 |
|  | Cu | 1.306996 | -0.63744 | 0.962348 |
|  | Cu | -1.28965 | 0.639122 | 0.881704 |
|  | O | -0.89547 | 0.563306 | 2.584359 |
|  | O | 1.182469 | -0.77845 | 2.800516 |
|  | H | -0.14259 | -0.08657 | 2.875543 |
|  | H | 1.759779 | -0.11243 | 3.261199 |

Figure 4 (Cu2-N8/Gr)

| Sub Figure | Symbol | X | Y | Z |
| --- | --- | --- | --- | --- |
| (a) | C | 5.532288 | -1.26481 | 0.800863 |
|  | C | 4.847923 | -0.02811 | 0.527841 |
|  | C | 3.461272 | -0.01665 | 0.171349 |
|  | C | 2.763539 | -1.26165 | 0.023805 |
|  | C | 3.527158 | -2.49478 | 0.1474 |
|  | C | 4.845461 | -2.47268 | 0.571368 |
|  | C | 2.825929 | 1.246636 | -0.10918 |
|  | C | 0.951627 | 2.542221 | -0.68372 |
|  | C | -0.44995 | 2.596091 | -0.74387 |
|  | C | -0.48211 | -2.52667 | -0.94004 |
|  | C | -3.15167 | -2.51548 | -0.71241 |
|  | C | -4.39956 | -2.44159 | -0.11908 |
|  | C | -2.54252 | -3.72021 | -1.15074 |
|  | C | -5.88851 | 1.212404 | 1.406917 |
|  | C | -5.87802 | -1.22297 | 1.225229 |
|  | C | -6.4203 | -0.03671 | 1.692224 |
|  | C | -1.17058 | -3.75253 | -1.23281 |
|  | C | 2.917516 | -3.72149 | -0.24891 |
|  | C | 1.62257 | -3.74133 | -0.69537 |
|  | C | 0.881782 | -2.51416 | -0.65504 |
|  | C | 0.945141 | -4.94414 | -1.13614 |
|  | C | -0.38342 | -4.95167 | -1.40614 |
|  | C | -4.40989 | 2.568243 | 0.178201 |
|  | C | -3.10905 | 2.654575 | -0.32501 |
|  | C | -2.47929 | 3.859266 | -0.71518 |
|  | C | -1.12152 | 3.851298 | -0.93307 |
|  | H | -6.37483 | -2.16456 | 1.454421 |
|  | H | -5.07324 | -3.29218 | -0.09979 |
|  | H | -3.14022 | -4.61642 | -1.26526 |
|  | H | -0.89111 | -5.86574 | -1.69177 |
|  | H | -6.40206 | 2.106648 | 1.754818 |
|  | H | -7.32338 | -0.08505 | 2.287615 |
|  | C | 5.611877 | 1.191244 | 0.592028 |
|  | C | 4.997128 | 2.392745 | 0.182304 |
|  | C | 6.962032 | 1.153683 | 1.002646 |
|  | C | 3.661895 | 2.442656 | -0.17718 |
|  | C | 3.068663 | 3.662957 | -0.61523 |
|  | C | 1.724914 | 3.72557 | -0.88678 |
|  | C | 1.031554 | 4.95131 | -1.23138 |
|  | C | -0.32283 | 5.016842 | -1.2369 |
|  | C | 6.88343 | -1.24825 | 1.216611 |
|  | C | 7.578861 | -0.05074 | 1.327323 |
|  | H | 7.51463 | 2.084923 | 1.053053 |
|  | H | 5.592493 | 3.29826 | 0.13402 |
|  | H | 3.690976 | 4.547723 | -0.69168 |
|  | H | 1.629102 | 5.832295 | -1.43679 |
|  | H | -0.83598 | 5.949272 | -1.44185 |
|  | H | -3.05654 | 4.774653 | -0.74906 |
|  | H | -5.10125 | 3.402561 | 0.152546 |
|  | H | 1.52921 | -5.85502 | -1.20525 |
|  | H | 3.504264 | -4.63256 | -0.21014 |
|  | H | 5.379017 | -3.41057 | 0.684049 |
|  | H | 7.371646 | -2.19163 | 1.432686 |
|  | H | 8.614187 | -0.05595 | 1.648126 |
|  | N | 1.436195 | -1.30866 | -0.29733 |
|  | N | -1.12268 | -1.32086 | -0.87618 |
|  | N | -2.45824 | -1.29878 | -0.76619 |
|  | N | -4.74537 | -1.27174 | 0.480439 |
|  | N | 1.49018 | 1.341258 | -0.31191 |
|  | N | -1.09747 | 1.421616 | -0.58605 |
|  | N | -2.45313 | 1.439415 | -0.3838 |
|  | N | -4.73905 | 1.374795 | 0.710836 |
|  | Cu | 0.08281 | 0.011927 | 0.018594 |
|  | Cu | -3.34224 | 0.034094 | 0.456376 |
|  | O | -0.74845 | -0.02451 | 1.716451 |
|  | O | -2.11112 | -0.55642 | 1.778021 |
| (b) | C | 5.632577 | -1.22879 | 0.653376 |
|  | C | 4.904474 | 0.001483 | 0.510332 |
|  | C | 3.50237 | 0.001601 | 0.191365 |
|  | C | 2.836854 | -1.2538 | -0.03044 |
|  | C | 3.646299 | -2.46698 | -0.05473 |
|  | C | 4.978451 | -2.43542 | 0.324115 |
|  | C | 2.834742 | 1.259253 | -0.0137 |
|  | C | 0.940455 | 2.538449 | -0.60982 |
|  | C | -0.45221 | 2.573578 | -0.77382 |
|  | C | -0.4506 | -2.57365 | -0.78134 |
|  | C | -3.14406 | -2.59496 | -0.53558 |
|  | C | -4.43624 | -2.51275 | -0.05511 |
|  | C | -2.50452 | -3.78266 | -0.99015 |
|  | C | -5.99823 | 1.220175 | 1.120605 |
|  | C | -5.98875 | -1.21698 | 1.148388 |
|  | C | -6.5527 | 0.00463 | 1.494153 |
|  | C | -1.13684 | -3.80018 | -1.09744 |
|  | C | 3.041113 | -3.68438 | -0.48483 |
|  | C | 1.703512 | -3.72804 | -0.80069 |
|  | C | 0.944773 | -2.53388 | -0.62848 |
|  | C | 1.013917 | -4.92937 | -1.23196 |
|  | C | -0.33457 | -4.96622 | -1.37998 |
|  | C | -4.44543 | 2.499498 | -0.10107 |
|  | C | -3.14829 | 2.584362 | -0.56734 |
|  | C | -2.5082 | 3.773065 | -1.01918 |
|  | C | -1.13868 | 3.796982 | -1.09979 |
|  | H | -6.53757 | -2.13467 | 1.359373 |
|  | H | -5.13133 | -3.34342 | -0.11459 |
|  | H | -3.09732 | -4.67516 | -1.15089 |
|  | H | -0.83903 | -5.88079 | -1.66978 |
|  | H | -6.55301 | 2.139234 | 1.308254 |
|  | H | -7.50101 | 0.006976 | 2.015873 |
|  | C | 5.632211 | 1.232034 | 0.659951 |
|  | C | 4.977959 | 2.439894 | 0.333273 |
|  | C | 6.988836 | 1.206923 | 1.052276 |
|  | C | 3.644049 | 2.472177 | -0.04064 |
|  | C | 3.037181 | 3.689408 | -0.46773 |
|  | C | 1.700495 | 3.732249 | -0.78699 |
|  | C | 1.011075 | 4.931161 | -1.22389 |
|  | C | -0.33695 | 4.964333 | -1.37812 |
|  | C | 6.989864 | -1.2046 | 1.046749 |
|  | C | 7.64916 | 0.000443 | 1.260446 |
|  | H | 7.511761 | 2.148972 | 1.172482 |
|  | H | 5.543646 | 3.365473 | 0.348841 |
|  | H | 3.65022 | 4.581378 | -0.53535 |
|  | H | 1.608032 | 5.819017 | -1.39991 |
|  | H | -0.8418 | 5.876497 | -1.67484 |
|  | H | -3.1031 | 4.660645 | -1.19789 |
|  | H | -5.14553 | 3.32425 | -0.18342 |
|  | H | 1.611796 | -5.8169 | -1.40658 |
|  | H | 3.654824 | -4.57575 | -0.55314 |
|  | H | 5.542744 | -3.36177 | 0.343811 |
|  | H | 7.512784 | -2.14711 | 1.16326 |
|  | H | 8.689384 | 0.000162 | 1.565259 |
|  | N | 1.495915 | -1.3317 | -0.25921 |
|  | N | -1.09711 | -1.40897 | -0.57125 |
|  | N | -2.44191 | -1.38059 | -0.47239 |
|  | N | -4.80026 | -1.32794 | 0.519609 |
|  | N | 1.491416 | 1.339897 | -0.22485 |
|  | N | -1.09676 | 1.399492 | -0.58451 |
|  | N | -2.44317 | 1.370337 | -0.49191 |
|  | N | -4.80471 | 1.326209 | 0.496346 |
|  | Cu | 0.103498 | -0.00297 | 0.028264 |
|  | Cu | -3.38149 | 0.003083 | 0.492911 |
|  | O | -0.81744 | -0.07915 | 1.882133 |
|  | O | -2.34867 | 0.033562 | 2.12673 |
|  | H | -0.51328 | 0.413025 | 2.687958 |
| (c) | C | 5.698938 | -1.24271 | 0.521196 |
|  | C | 4.961237 | -0.00854 | 0.38764 |
|  | C | 3.56144 | -0.00846 | 0.093732 |
|  | C | 2.886925 | -1.27004 | -0.07375 |
|  | C | 3.688374 | -2.48668 | -0.07242 |
|  | C | 5.037411 | -2.45231 | 0.240741 |
|  | C | 2.884671 | 1.249226 | -0.09085 |
|  | C | 0.975791 | 2.515043 | -0.61612 |
|  | C | -0.42653 | 2.552376 | -0.7319 |
|  | C | -0.43715 | -2.56407 | -0.64755 |
|  | C | -3.13052 | -2.57454 | -0.49893 |
|  | C | -4.49861 | -2.49922 | -0.23803 |
|  | C | -2.48312 | -3.78247 | -0.83514 |
|  | C | -6.14148 | 1.225682 | 0.877763 |
|  | C | -6.20227 | -1.21626 | 0.729269 |
|  | C | -6.76613 | 0.007386 | 1.069315 |
|  | C | -1.11418 | -3.80396 | -0.90748 |
|  | C | 3.060704 | -3.71029 | -0.43522 |
|  | C | 1.718194 | -3.7496 | -0.71423 |
|  | C | 0.971764 | -2.53749 | -0.55941 |
|  | C | 1.016468 | -4.96697 | -1.06116 |
|  | C | -0.33368 | -4.99337 | -1.15418 |
|  | C | -4.44182 | 2.516341 | -0.07959 |
|  | C | -3.10651 | 2.588052 | -0.46828 |
|  | C | -2.46719 | 3.791108 | -0.83475 |
|  | C | -1.10196 | 3.797245 | -0.97536 |
|  | H | -6.77402 | -2.12937 | 0.890869 |
|  | H | -5.14964 | -3.35529 | -0.38303 |
|  | H | -3.07399 | -4.68049 | -0.96547 |
|  | H | -0.86225 | -5.91067 | -1.386 |
|  | H | -6.66009 | 2.143968 | 1.149717 |
|  | H | -7.76304 | 0.006537 | 1.493288 |
|  | C | 5.693079 | 1.22686 | 0.535554 |
|  | C | 5.025263 | 2.436036 | 0.269767 |
|  | C | 7.061697 | 1.199598 | 0.884944 |
|  | C | 3.681565 | 2.467808 | -0.06552 |
|  | C | 3.06152 | 3.691388 | -0.44096 |
|  | C | 1.725607 | 3.729596 | -0.74715 |
|  | C | 1.032439 | 4.947657 | -1.10817 |
|  | C | -0.31624 | 4.981687 | -1.223 |
|  | C | 7.068407 | -1.21332 | 0.867695 |
|  | C | 7.73029 | -0.00641 | 1.059817 |
|  | H | 7.584558 | 2.141628 | 1.003459 |
|  | H | 5.585237 | 3.364627 | 0.298668 |
|  | H | 3.661932 | 4.593792 | -0.4749 |
|  | H | 1.624619 | 5.845278 | -1.246 |
|  | H | -0.83703 | 5.903652 | -1.45383 |
|  | H | -3.05079 | 4.699667 | -0.91936 |
|  | H | -5.08658 | 3.388452 | -0.12587 |
|  | H | 1.603069 | -5.86525 | -1.21675 |
|  | H | 3.660282 | -4.61296 | -0.47951 |
|  | H | 5.604565 | -3.377 | 0.243533 |
|  | H | 7.596375 | -2.15417 | 0.972145 |
|  | H | 8.779985 | -0.0054 | 1.330477 |
|  | N | 1.539194 | -1.34116 | -0.25321 |
|  | N | -1.07325 | -1.38883 | -0.4526 |
|  | N | -2.43937 | -1.37578 | -0.38072 |
|  | N | -4.94964 | -1.32632 | 0.236367 |
|  | N | 1.54272 | 1.312043 | -0.32152 |
|  | N | -1.08032 | 1.377394 | -0.55745 |
|  | N | -2.42533 | 1.375047 | -0.44481 |
|  | N | -4.89345 | 1.326879 | 0.351132 |
|  | Cu | 0.132571 | 0.00623 | 0.167435 |
|  | Cu | -3.46989 | -0.02069 | 0.463001 |
|  | O | -0.24375 | 0.251172 | 1.973616 |
|  | O | -2.88263 | -0.00921 | 2.176245 |
|  | H | -1.85836 | 0.116606 | 2.196079 |
|  | H | 0.218068 | -0.36306 | 2.600769 |
| (d) | C | 5.488858 | -1.22281 | 0.806857 |
|  | C | 4.768548 | 0.004698 | 0.638063 |
|  | C | 3.380163 | 0.003539 | 0.263769 |
|  | C | 2.722003 | -1.25762 | 0.016602 |
|  | C | 3.539576 | -2.46453 | 0.00509 |
|  | C | 4.853124 | -2.42969 | 0.441265 |
|  | C | 2.729082 | 1.254429 | 0.016145 |
|  | C | 0.872592 | 2.517755 | -0.70446 |
|  | C | -0.52129 | 2.570632 | -0.9075 |
|  | C | -0.50465 | -2.57629 | -0.90852 |
|  | C | -3.16861 | -2.61777 | -0.49284 |
|  | C | -4.37157 | -2.53437 | 0.1975 |
|  | C | -2.5602 | -3.8032 | -1.01917 |
|  | C | -5.78453 | 1.216483 | 1.534333 |
|  | C | -5.77248 | -1.23274 | 1.566285 |
|  | C | -6.27963 | -0.01574 | 1.977979 |
|  | C | -1.20245 | -3.80581 | -1.20451 |
|  | C | 2.958229 | -3.68247 | -0.47456 |
|  | C | 1.643304 | -3.72115 | -0.85703 |
|  | C | 0.867563 | -2.53108 | -0.69828 |
|  | C | 0.961974 | -4.91282 | -1.33896 |
|  | C | -0.38436 | -4.95981 | -1.50725 |
|  | C | -4.38643 | 2.551099 | 0.194778 |
|  | C | -3.18466 | 2.632708 | -0.4723 |
|  | C | -2.56885 | 3.808153 | -1.00119 |
|  | C | -1.20942 | 3.804419 | -1.19744 |
|  | H | -6.33759 | -2.14139 | 1.776658 |
|  | H | -5.04937 | -3.37887 | 0.275975 |
|  | H | -3.15585 | -4.69867 | -1.1495 |
|  | H | -0.87449 | -5.87598 | -1.81652 |
|  | H | -6.38949 | 2.108039 | 1.705903 |
|  | H | -7.18725 | -0.01252 | 2.56874 |
|  | C | 5.488915 | 1.236372 | 0.804825 |
|  | C | 4.857789 | 2.434047 | 0.436242 |
|  | C | 6.837873 | 1.216523 | 1.253945 |
|  | C | 3.529681 | 2.460545 | -0.00127 |
|  | C | 2.956128 | 3.669215 | -0.47846 |
|  | C | 1.6345 | 3.707646 | -0.8731 |
|  | C | 0.957855 | 4.900439 | -1.35002 |
|  | C | -0.39178 | 4.953298 | -1.50558 |
|  | C | 6.830205 | -1.19424 | 1.254724 |
|  | C | 7.485214 | 0.013881 | 1.493144 |
|  | H | 7.353015 | 2.160703 | 1.38945 |
|  | H | 5.419839 | 3.361639 | 0.463666 |
|  | H | 3.573107 | 4.559927 | -0.52385 |
|  | H | 1.564589 | 5.780131 | -1.53443 |
|  | H | -0.88015 | 5.872545 | -1.80805 |
|  | H | -3.15611 | 4.711026 | -1.12028 |
|  | H | -5.07516 | 3.385879 | 0.266974 |
|  | H | 1.566476 | -5.79487 | -1.52068 |
|  | H | 3.580345 | -4.5689 | -0.52848 |
|  | H | 5.422261 | -3.3528 | 0.472461 |
|  | H | 7.349908 | -2.1356 | 1.39371 |
|  | H | 8.512197 | 0.010586 | 1.839557 |
|  | N | 1.390837 | -1.33342 | -0.25246 |
|  | N | -1.16135 | -1.40593 | -0.69776 |
|  | N | -2.47214 | -1.41461 | -0.49192 |
|  | N | -4.63877 | -1.35677 | 0.826225 |
|  | N | 1.382216 | 1.335392 | -0.25097 |
|  | N | -1.16656 | 1.421273 | -0.67227 |
|  | N | -2.48171 | 1.407838 | -0.48875 |
|  | N | -4.65876 | 1.353122 | 0.818862 |
|  | Cu | -0.02054 | -0.00255 | 0.013327 |
|  | Cu | -3.26728 | 0.004487 | 0.552198 |
|  | O | -1.42691 | -0.00335 | 1.4979 |
|  | H | -1.26421 | -0.03099 | 2.473951 |
| (e) | C | 5.781669 | -1.11782 | 0.201061 |
|  | C | 4.991936 | 0.075227 | 0.329601 |
|  | C | 3.566533 | 0.048958 | 0.123068 |
|  | C | 2.937584 | -1.18696 | -0.23674 |
|  | C | 3.769676 | -2.37374 | -0.38326 |
|  | C | 5.135381 | -2.3126 | -0.16804 |
|  | C | 2.82282 | 1.277901 | 0.226682 |
|  | C | 0.86499 | 2.538633 | -0.19876 |
|  | C | -0.4733 | 2.485766 | -0.57329 |
|  | C | -0.3574 | -2.61184 | -0.25571 |
|  | C | -3.05133 | -2.65349 | 0.119605 |
|  | C | -4.41709 | -2.56326 | 0.252719 |
|  | C | -2.39468 | -3.8694 | -0.20237 |
|  | C | -6.21541 | 1.266667 | 0.536572 |
|  | C | -6.2035 | -1.14497 | 0.798395 |
|  | C | -6.83145 | 0.094698 | 0.907368 |
|  | C | -1.04529 | -3.87116 | -0.40158 |
|  | C | 3.149892 | -3.62272 | -0.69499 |
|  | C | 1.785942 | -3.7346 | -0.69127 |
|  | C | 1.020386 | -2.54737 | -0.47837 |
|  | C | 1.074514 | -4.98546 | -0.86431 |
|  | C | -0.27263 | -5.04421 | -0.73979 |
|  | C | -4.50399 | 2.322309 | -0.67945 |
|  | C | -3.13221 | 2.357092 | -0.94928 |
|  | C | -2.49098 | 3.587142 | -1.23013 |
|  | C | -1.15864 | 3.69768 | -0.93823 |
|  | H | -6.75609 | -2.04576 | 1.060893 |
|  | H | -5.06195 | -3.43214 | 0.301327 |
|  | H | -2.98767 | -4.76852 | -0.31666 |
|  | H | -0.80501 | -5.97991 | -0.86804 |
|  | H | -6.77689 | 2.196493 | 0.501005 |
|  | H | -7.85742 | 0.12781 | 1.250716 |
|  | C | 5.672843 | 1.297736 | 0.657316 |
|  | C | 4.932094 | 2.500165 | 0.636606 |
|  | C | 7.060578 | 1.287504 | 0.908367 |
|  | C | 3.577241 | 2.521341 | 0.374108 |
|  | C | 2.896676 | 3.762789 | 0.181274 |
|  | C | 1.578539 | 3.7796 | -0.18267 |
|  | C | 0.870389 | 4.985148 | -0.5544 |
|  | C | -0.43068 | 4.943492 | -0.92706 |
|  | C | 7.174173 | -1.07809 | 0.452588 |
|  | C | 7.795898 | 0.108558 | 0.812809 |
|  | H | 7.54921 | 2.221649 | 1.161151 |
|  | H | 5.455381 | 3.440457 | 0.773852 |
|  | H | 3.463247 | 4.683965 | 0.257064 |
|  | H | 1.417032 | 5.921095 | -0.53699 |
|  | H | -0.96176 | 5.843954 | -1.21363 |
|  | H | -3.09102 | 4.446808 | -1.50011 |
|  | H | -5.191 | 3.069301 | -1.05978 |
|  | H | 1.654821 | -5.87292 | -1.0893 |
|  | H | 3.778971 | -4.48958 | -0.86274 |
|  | H | 5.720215 | -3.22279 | -0.24847 |
|  | H | 7.744614 | -1.99476 | 0.355218 |
|  | H | 8.862438 | 0.122259 | 1.00732 |
|  | N | 1.579788 | -1.28798 | -0.41899 |
|  | N | -0.94308 | -1.46459 | 0.19764 |
|  | N | -2.34746 | -1.46489 | 0.391209 |
|  | N | -4.93372 | -1.29468 | 0.373226 |
|  | N | 1.462143 | 1.323528 | 0.125148 |
|  | N | -1.04085 | 1.235076 | -0.68325 |
|  | N | -2.46614 | 1.167437 | -0.71722 |
|  | N | -4.91025 | 1.32544 | 0.128467 |
|  | Cu | 0.199161 | -0.04195 | -0.14011 |
|  | Cu | -3.56046 | 0.00769 | 0.293317 |
|  | O | -2.70009 | 0.306953 | 2.298743 |
|  | H | -2.22433 | -0.56625 | 2.232091 |
|  | H | -2.54463 | 0.736911 | 3.177827 |

Figure 7 (Cu-N4/Gr)

| Sub Figure | Symbol | X | Y | Z |
| --- | --- | --- | --- | --- |
| (a) | C | 5.564519 | -1.18823 | -0.1133 |
|  | C | 4.8143 | 0.045947 | -0.08489 |
|  | C | 3.384163 | 0.045992 | -0.09351 |
|  | C | 2.689691 | -1.21656 | -0.16083 |
|  | C | 3.471251 | -2.43588 | -0.22836 |
|  | C | 4.862868 | -2.3968 | -0.19112 |
|  | C | 2.683969 | 1.310768 | -0.08417 |
|  | C | 0.705909 | 2.599926 | -0.13222 |
|  | C | -0.70579 | 2.599934 | -0.13247 |
|  | C | -2.68967 | -1.21656 | -0.16061 |
|  | C | -0.71067 | -2.49602 | -0.25326 |
|  | C | -3.3841 | 0.046023 | -0.09361 |
|  | C | -3.47125 | -2.4359 | -0.22772 |
|  | C | -4.81424 | 0.046004 | -0.08495 |
|  | C | -4.86286 | -2.39679 | -0.19029 |
|  | C | -2.79394 | -3.67793 | -0.32052 |
|  | C | -5.56384 | 1.279954 | -0.06162 |
|  | C | -5.56447 | -1.18817 | -0.11285 |
|  | C | -6.97841 | 1.250808 | -0.02692 |
|  | C | -6.98201 | -1.1601 | -0.07588 |
|  | C | -7.66998 | 0.044163 | -0.02819 |
|  | C | -1.42067 | -3.73226 | -0.33996 |
|  | C | 2.793936 | -3.67789 | -0.32123 |
|  | C | 1.420651 | -3.73221 | -0.34031 |
|  | C | 0.710675 | -2.496 | -0.25337 |
|  | C | 0.676067 | -4.96892 | -0.42855 |
|  | C | -0.67608 | -4.96894 | -0.42835 |
|  | C | -2.68388 | 1.310797 | -0.08461 |
|  | C | -4.85859 | 2.491269 | -0.09727 |
|  | C | -3.46927 | 2.532325 | -0.1243 |
|  | C | -2.79288 | 3.778966 | -0.1851 |
|  | C | -1.42087 | 3.835095 | -0.20489 |
|  | H | -7.51728 | -2.10236 | -0.09003 |
|  | H | -5.41572 | -3.32937 | -0.22158 |
|  | H | -3.3797 | -4.58921 | -0.37132 |
|  | H | -1.23548 | -5.89531 | -0.48935 |
|  | H | -7.51409 | 2.192701 | -0.00356 |
|  | H | -8.75354 | 0.045145 | 0.000847 |
|  | C | 5.563928 | 1.279871 | -0.06112 |
|  | C | 4.858701 | 2.491207 | -0.09639 |
|  | C | 6.978506 | 1.250689 | -0.02643 |
|  | C | 3.469383 | 2.532292 | -0.12352 |
|  | C | 2.79302 | 3.778952 | -0.18424 |
|  | C | 1.421016 | 3.835087 | -0.2044 |
|  | C | 0.676308 | 5.073045 | -0.27661 |
|  | C | -0.67614 | 5.073048 | -0.27686 |
|  | C | 6.982046 | -1.1602 | -0.07639 |
|  | C | 7.670051 | 0.044037 | -0.02821 |
|  | H | 7.514187 | 2.192566 | -0.00268 |
|  | H | 5.411726 | 3.42418 | -0.10513 |
|  | H | 3.380739 | 4.689701 | -0.21714 |
|  | H | 1.23584 | 6.000028 | -0.3269 |
|  | H | -1.23564 | 6.000034 | -0.32736 |
|  | H | -3.38058 | 4.689717 | -0.21829 |
|  | H | -5.4116 | 3.424247 | -0.10631 |
|  | H | 1.235485 | -5.89526 | -0.48973 |
|  | H | 3.379677 | -4.58916 | -0.37229 |
|  | H | 5.415702 | -3.32938 | -0.22277 |
|  | H | 7.517312 | -2.10246 | -0.09097 |
|  | H | 8.753602 | 0.044979 | 0.0008 |
|  | N | 1.329364 | -1.28975 | -0.17556 |
|  | N | -1.32937 | -1.28981 | -0.17555 |
|  | N | 1.324804 | 1.384382 | -0.06034 |
|  | N | -1.32471 | 1.384401 | -0.0608 |
|  | Cu | -5.7E-05 | 0.026955 | 0.353233 |
|  | O | -0.00073 | -0.26734 | 2.307576 |
|  | O | -0.00023 | -1.62792 | 2.546428 |
| (b) | C | 5.544104 | -1.18811 | 0.02238 |
|  | C | 4.798212 | 0.045942 | -0.00262 |
|  | C | 3.371707 | 0.047598 | -0.11275 |
|  | C | 2.679922 | -1.21436 | -0.19711 |
|  | C | 3.462926 | -2.43924 | -0.198 |
|  | C | 4.843881 | -2.40306 | -0.09261 |
|  | C | 2.675541 | 1.309753 | -0.12968 |
|  | C | 0.699611 | 2.589051 | -0.28439 |
|  | C | -0.70736 | 2.589287 | -0.28716 |
|  | C | -2.68137 | -1.21518 | -0.18636 |
|  | C | -0.70668 | -2.48321 | -0.40063 |
|  | C | -3.37474 | 0.044817 | -0.10546 |
|  | C | -3.46244 | -2.44008 | -0.18513 |
|  | C | -4.80069 | 0.042185 | 0.003788 |
|  | C | -4.84375 | -2.40572 | -0.07899 |
|  | C | -2.78614 | -3.68108 | -0.35403 |
|  | C | -5.54705 | 1.273949 | 0.071541 |
|  | C | -5.54489 | -1.19232 | 0.033338 |
|  | C | -6.95487 | 1.242559 | 0.184293 |
|  | C | -6.95411 | -1.1676 | 0.14821 |
|  | C | -7.64389 | 0.035489 | 0.226581 |
|  | C | -1.42275 | -3.72417 | -0.48361 |
|  | C | 2.78491 | -3.68082 | -0.3654 |
|  | C | 1.42042 | -3.72482 | -0.48735 |
|  | C | 0.703119 | -2.48423 | -0.40156 |
|  | C | 0.67452 | -4.95698 | -0.63324 |
|  | C | -0.67738 | -4.9562 | -0.63137 |
|  | C | -2.68074 | 1.308767 | -0.12869 |
|  | C | -4.84728 | 2.492273 | -0.00589 |
|  | C | -3.46621 | 2.532868 | -0.10118 |
|  | C | -2.7909 | 3.78003 | -0.23191 |
|  | C | -1.42649 | 3.829376 | -0.35033 |
|  | H | -7.48797 | -2.11068 | 0.173277 |
|  | H | -5.40105 | -3.33596 | -0.1095 |
|  | H | -3.37032 | -4.59477 | -0.37241 |
|  | H | -1.23674 | -5.88073 | -0.71884 |
|  | H | -7.49103 | 2.183207 | 0.237271 |
|  | H | -8.72388 | 0.03381 | 0.316754 |
|  | C | 5.542478 | 1.278684 | 0.071832 |
|  | C | 4.840243 | 2.495586 | 0.001159 |
|  | C | 6.950969 | 1.249201 | 0.185274 |
|  | C | 3.458739 | 2.534334 | -0.09509 |
|  | C | 2.782427 | 3.781443 | -0.22217 |
|  | C | 1.418277 | 3.82996 | -0.34266 |
|  | C | 0.671965 | 5.064887 | -0.46066 |
|  | C | -0.68028 | 5.064582 | -0.46517 |
|  | C | 6.952572 | -1.16122 | 0.137711 |
|  | C | 7.640805 | 0.043262 | 0.221632 |
|  | H | 7.485469 | 2.190498 | 0.24281 |
|  | H | 5.397958 | 3.426037 | -0.00423 |
|  | H | 3.368269 | 4.694175 | -0.22037 |
|  | H | 1.230761 | 5.991577 | -0.52568 |
|  | H | -1.23899 | 5.991086 | -0.53355 |
|  | H | -3.37739 | 4.692358 | -0.23251 |
|  | H | -5.40701 | 3.421445 | -0.01747 |
|  | H | 1.23314 | -5.88173 | -0.7229 |
|  | H | 3.369555 | -4.59411 | -0.39045 |
|  | H | 5.402849 | -3.33222 | -0.1265 |
|  | H | 7.488452 | -2.1033 | 0.157842 |
|  | H | 8.720881 | 0.042184 | 0.31146 |
|  | N | 1.323836 | -1.27672 | -0.28529 |
|  | N | -1.32547 | -1.27695 | -0.2772 |
|  | N | 1.316672 | 1.374757 | -0.19193 |
|  | N | -1.32412 | 1.375505 | -0.18905 |
|  | Cu | -0.00353 | 0.026007 | 0.318624 |
|  | O | 0.03103 | -0.083 | 2.1809 |
|  | O | -0.05217 | -1.5657 | 2.484505 |
|  | H | 0.867796 | -1.69853 | 2.85643 |
| (c) | C | 5.553224 | -1.23523 | 0.068051 |
|  | C | 4.803138 | 0.000011 | 0.050177 |
|  | C | 3.375785 | 0.000009 | -0.00147 |
|  | C | 2.68079 | -1.2652 | -0.05975 |
|  | C | 3.465649 | -2.4842 | -0.11712 |
|  | C | 4.854342 | -2.44448 | -0.03443 |
|  | C | 2.68078 | 1.26521 | -0.05971 |
|  | C | 0.707789 | 2.546151 | -0.21714 |
|  | C | -0.7078 | 2.546152 | -0.21716 |
|  | C | -2.68078 | -1.2652 | -0.05976 |
|  | C | -0.70779 | -2.54613 | -0.21719 |
|  | C | -3.37578 | 0.000008 | -0.00149 |
|  | C | -3.46564 | -2.4842 | -0.11706 |
|  | C | -4.80313 | 0.000007 | 0.05016 |
|  | C | -4.85433 | -2.44448 | -0.03436 |
|  | C | -2.79318 | -3.72614 | -0.25682 |
|  | C | -5.55322 | 1.235248 | 0.068019 |
|  | C | -5.55321 | -1.23524 | 0.068078 |
|  | C | -6.9668 | 1.206027 | 0.158211 |
|  | C | -6.9668 | -1.20602 | 0.158281 |
|  | C | -7.65451 | 0.000004 | 0.210363 |
|  | C | -1.4222 | -3.77981 | -0.32295 |
|  | C | 2.793188 | -3.72613 | -0.25689 |
|  | C | 1.422211 | -3.7798 | -0.32298 |
|  | C | 0.707796 | -2.54613 | -0.2172 |
|  | C | 0.676347 | -5.0121 | -0.4573 |
|  | C | -0.67634 | -5.01211 | -0.45727 |
|  | C | -2.68078 | 1.265212 | -0.05976 |
|  | C | -4.85434 | 2.444491 | -0.03446 |
|  | C | -3.46564 | 2.484214 | -0.11712 |
|  | C | -2.79319 | 3.726153 | -0.25686 |
|  | C | -1.42221 | 3.779829 | -0.32294 |
|  | H | -7.50238 | -2.14794 | 0.179966 |
|  | H | -5.40911 | -3.37617 | -0.05604 |
|  | H | -3.38203 | -4.63541 | -0.30658 |
|  | H | -1.23497 | -5.93673 | -0.54615 |
|  | H | -7.50239 | 2.147945 | 0.179837 |
|  | H | -8.73611 | 0.000003 | 0.281739 |
|  | C | 5.55322 | 1.235254 | 0.068066 |
|  | C | 4.854331 | 2.444494 | -0.0344 |
|  | C | 6.966806 | 1.206037 | 0.158262 |
|  | C | 3.465637 | 2.484213 | -0.11707 |
|  | C | 2.79318 | 3.726152 | -0.25682 |
|  | C | 1.422204 | 3.779826 | -0.32292 |
|  | C | 0.676343 | 5.012126 | -0.45724 |
|  | C | -0.67634 | 5.012128 | -0.45725 |
|  | C | 6.966808 | -1.20601 | 0.158253 |
|  | C | 7.654511 | 0.000015 | 0.210378 |
|  | H | 7.502389 | 2.147958 | 0.179915 |
|  | H | 5.409108 | 3.376182 | -0.05612 |
|  | H | 3.382037 | 4.635422 | -0.3066 |
|  | H | 1.234974 | 5.936754 | -0.54611 |
|  | H | -1.23497 | 5.936758 | -0.54613 |
|  | H | -3.38204 | 4.635423 | -0.30666 |
|  | H | -5.40912 | 3.376175 | -0.05619 |
|  | H | 1.234976 | -5.93673 | -0.54619 |
|  | H | 3.38204 | -4.63541 | -0.30668 |
|  | H | 5.409123 | -3.37616 | -0.05615 |
|  | H | 7.502396 | -2.14793 | 0.179902 |
|  | H | 8.736111 | 0.000016 | 0.281755 |
|  | N | 1.321389 | -1.33939 | -0.08577 |
|  | N | -1.32138 | -1.33939 | -0.08579 |
|  | N | 1.321367 | 1.33941 | -0.08569 |
|  | N | -1.32138 | 1.339411 | -0.08575 |
|  | Cu | 0.000041 | -2.4E-05 | 0.442442 |
|  | O | -0.00022 | -0.00026 | 2.319254 |
| (d) | C | -5.54009 | 1.233877 | 0.130199 |
|  | C | -4.7949 | -5.1E-05 | 0.077867 |
|  | C | -3.3704 | -7E-06 | -0.03983 |
|  | C | -2.67785 | 1.262997 | -0.09943 |
|  | C | -3.46093 | 2.487629 | -0.08776 |
|  | C | -4.84144 | 2.45003 | 0.024583 |
|  | C | -2.67763 | -1.26281 | -0.09829 |
|  | C | -0.70425 | -2.53506 | -0.30393 |
|  | C | 0.704307 | -2.53509 | -0.29981 |
|  | C | 2.680374 | 1.263093 | -0.09553 |
|  | C | 0.703916 | 2.535196 | -0.29874 |
|  | C | 3.373632 | 0.000111 | -0.04051 |
|  | C | 3.462804 | 2.487861 | -0.08278 |
|  | C | 4.799361 | 0.000151 | 0.076394 |
|  | C | 4.843607 | 2.450353 | 0.026924 |
|  | C | 2.785951 | 3.730837 | -0.24287 |
|  | C | 5.54409 | -1.23412 | 0.128086 |
|  | C | 5.543864 | 1.234468 | 0.129676 |
|  | C | 6.951701 | -1.20547 | 0.252424 |
|  | C | 6.951532 | 1.206002 | 0.254021 |
|  | C | 7.639627 | 0.000305 | 0.318999 |
|  | C | 1.42245 | 3.775175 | -0.37626 |
|  | C | -2.78577 | 3.730206 | -0.25359 |
|  | C | -1.42216 | 3.775411 | -0.38629 |
|  | C | -0.70458 | 2.535319 | -0.30452 |
|  | C | -0.67557 | 5.00737 | -0.52915 |
|  | C | 0.676641 | 5.007329 | -0.52425 |
|  | C | 2.680641 | -1.26309 | -0.09716 |
|  | C | 4.844081 | -2.4501 | 0.024047 |
|  | C | 3.463314 | -2.48778 | -0.08546 |
|  | C | 2.786409 | -3.73068 | -0.2459 |
|  | C | 1.42277 | -3.77504 | -0.37808 |
|  | H | 7.48674 | 2.147694 | 0.29595 |
|  | H | 5.40235 | 3.379961 | 0.006564 |
|  | H | 3.370621 | 4.64424 | -0.25609 |
|  | H | 1.235748 | 5.932269 | -0.60853 |
|  | H | 7.487119 | -2.1471 | 0.293126 |
|  | H | 8.719058 | 0.000315 | 0.416568 |
|  | C | -5.53984 | -1.23405 | 0.131671 |
|  | C | -4.84098 | -2.45008 | 0.027318 |
|  | C | -6.94738 | -1.20562 | 0.257392 |
|  | C | -3.46043 | -2.4875 | -0.08541 |
|  | C | -2.7853 | -3.7301 | -0.25087 |
|  | C | -1.42182 | -3.77525 | -0.38471 |
|  | C | -0.67538 | -5.00722 | -0.52823 |
|  | C | 0.676831 | -5.00721 | -0.52511 |
|  | C | -6.94753 | 1.205265 | 0.255885 |
|  | C | -7.63588 | -0.00024 | 0.323299 |
|  | H | -7.48231 | -2.14742 | 0.300776 |
|  | H | -5.40028 | -3.37935 | 0.006296 |
|  | H | -3.37035 | -4.64332 | -0.26314 |
|  | H | -1.23368 | -5.93242 | -0.61519 |
|  | H | 1.235815 | -5.93217 | -0.60996 |
|  | H | 3.371078 | -4.64407 | -0.25982 |
|  | H | 5.40305 | -3.37955 | 0.002751 |
|  | H | -1.23374 | 5.932571 | -0.61695 |
|  | H | -3.37087 | 4.643374 | -0.26673 |
|  | H | -5.40097 | 3.37913 | 0.002457 |
|  | H | -7.48271 | 2.146983 | 0.298092 |
|  | H | -8.71508 | -0.00024 | 0.422787 |
|  | N | -1.32272 | 1.327429 | -0.18273 |
|  | N | 1.321315 | 1.325511 | -0.16941 |
|  | N | -1.32232 | -1.32716 | -0.1822 |
|  | N | 1.321886 | -1.32554 | -0.17106 |
|  | Cu | -0.00331 | 0.000051 | 0.385065 |
|  | O | -0.1212 | -0.00364 | 2.223821 |
|  | H | 0.769824 | 0.010362 | 2.665565 |
| (e) | C | 5.564706 | -1.23266 | 0.065183 |
|  | C | 4.819515 | 0.000138 | 0.049973 |
|  | C | 3.385819 | 0.000074 | 0.000136 |
|  | C | 2.688525 | -1.26372 | -0.05776 |
|  | C | 3.474035 | -2.48239 | -0.13495 |
|  | C | 4.860563 | -2.44266 | -0.04972 |
|  | C | 2.688393 | 1.264038 | -0.06081 |
|  | C | 0.702122 | 2.548217 | -0.22191 |
|  | C | -0.70194 | 2.548267 | -0.22002 |
|  | C | -2.68841 | -1.26403 | -0.0608 |
|  | C | -0.70213 | -2.54821 | -0.22191 |
|  | C | -3.38583 | -0.00006 | 0.000176 |
|  | C | -3.47403 | -2.48256 | -0.13978 |
|  | C | -4.81953 | -0.00012 | 0.049995 |
|  | C | -4.86069 | -2.4429 | -0.05485 |
|  | C | -2.79471 | -3.7239 | -0.30256 |
|  | C | -5.56472 | 1.232668 | 0.065221 |
|  | C | -5.56478 | -1.23306 | 0.062341 |
|  | C | -6.97911 | 1.205566 | 0.157933 |
|  | C | -6.97902 | -1.20608 | 0.155032 |
|  | C | -7.66829 | -0.00025 | 0.210963 |
|  | C | -1.42375 | -3.77516 | -0.36351 |
|  | C | 2.794681 | -3.72407 | -0.2971 |
|  | C | 1.423855 | -3.77521 | -0.36037 |
|  | C | 0.701933 | -2.54825 | -0.22006 |
|  | C | 0.676899 | -5.00389 | -0.52686 |
|  | C | -0.67658 | -5.00386 | -0.52837 |
|  | C | -2.68853 | 1.263727 | -0.05769 |
|  | C | -4.86057 | 2.442666 | -0.04966 |
|  | C | -3.47404 | 2.482398 | -0.13488 |
|  | C | -2.79469 | 3.724081 | -0.29703 |
|  | C | -1.42387 | 3.77522 | -0.36033 |
|  | H | -7.51408 | -2.14879 | 0.17081 |
|  | H | -5.41604 | -3.37424 | -0.09244 |
|  | H | -3.3826 | -4.63207 | -0.38047 |
|  | H | -1.23462 | -5.92599 | -0.64625 |
|  | H | -7.51409 | 2.148284 | 0.175942 |
|  | H | -8.75009 | -0.00038 | 0.281979 |
|  | C | 5.564768 | 1.233072 | 0.062355 |
|  | C | 4.860667 | 2.442914 | -0.05484 |
|  | C | 6.979006 | 1.206094 | 0.155075 |
|  | C | 3.474013 | 2.482564 | -0.13978 |
|  | C | 2.794694 | 3.723912 | -0.30258 |
|  | C | 1.42374 | 3.775169 | -0.36353 |
|  | C | 0.676567 | 5.003862 | -0.5284 |
|  | C | -0.67691 | 5.003895 | -0.52687 |
|  | C | 6.979099 | -1.20555 | 0.157918 |
|  | C | 7.668276 | 0.00027 | 0.210991 |
|  | H | 7.514058 | 2.148808 | 0.170886 |
|  | H | 5.416019 | 3.374254 | -0.09242 |
|  | H | 3.382586 | 4.632078 | -0.38051 |
|  | H | 1.234604 | 5.925991 | -0.64631 |
|  | H | -1.2352 | 5.926022 | -0.64359 |
|  | H | -3.38268 | 4.632287 | -0.37372 |
|  | H | -5.41589 | 3.374092 | -0.08573 |
|  | H | 1.235185 | -5.92602 | -0.64357 |
|  | H | 3.382658 | -4.63228 | -0.3738 |
|  | H | 5.415887 | -3.37408 | -0.08581 |
|  | H | 7.514079 | -2.14826 | 0.175912 |
|  | H | 8.750069 | 0.000398 | 0.282029 |
|  | N | 1.328097 | -1.33445 | -0.06316 |
|  | N | -1.32853 | -1.33461 | -0.06671 |
|  | N | 1.328506 | 1.334615 | -0.06673 |
|  | N | -1.32809 | 1.334469 | -0.06307 |
|  | Cu | -0.00004 | -4E-06 | 0.370061 |
|  | O | 0.000291 | -0.00011 | 2.452961 |
|  | H | -0.80432 | 0.080709 | 3.025563 |
|  | H | 0.804938 | -0.08154 | 3.025427 |
| (f) | C | 5.519249 | -1.18823 | 0.158891 |
|  | C | 4.778359 | 0.04607 | 0.101858 |
|  | C | 3.354102 | 0.045589 | -0.07018 |
|  | C | 2.662919 | -1.20896 | -0.18396 |
|  | C | 3.447519 | -2.43364 | -0.20724 |
|  | C | 4.821312 | -2.39999 | -0.03102 |
|  | C | 2.656378 | 1.299589 | -0.14229 |
|  | C | 0.677325 | 2.56941 | -0.41651 |
|  | C | -0.72215 | 2.565975 | -0.40757 |
|  | C | -2.68729 | -1.22399 | -0.16413 |
|  | C | -0.71014 | -2.48252 | -0.50091 |
|  | C | -3.38454 | 0.027049 | -0.04589 |
|  | C | -3.46627 | -2.45194 | -0.18736 |
|  | C | -4.80805 | 0.020131 | 0.131257 |
|  | C | -4.83974 | -2.42521 | -0.00817 |
|  | C | -2.7911 | -3.67883 | -0.46326 |
|  | C | -5.54941 | 1.251338 | 0.229785 |
|  | C | -5.54262 | -1.21781 | 0.187572 |
|  | C | -6.94837 | 1.216388 | 0.429166 |
|  | C | -6.94166 | -1.19731 | 0.388069 |
|  | C | -7.62675 | 0.005857 | 0.515097 |
|  | C | -1.43203 | -3.70925 | -0.65006 |
|  | C | 2.776546 | -3.66422 | -0.48071 |
|  | C | 1.416626 | -3.70143 | -0.66122 |
|  | C | 0.689037 | -2.4784 | -0.50819 |
|  | C | 0.671248 | -4.92075 | -0.90236 |
|  | C | -0.68196 | -4.92428 | -0.8972 |
|  | C | -2.69391 | 1.284616 | -0.11764 |
|  | C | -4.85293 | 2.468397 | 0.075485 |
|  | C | -3.47929 | 2.508639 | -0.10084 |
|  | C | -2.81042 | 3.748113 | -0.33376 |
|  | C | -1.45109 | 3.792538 | -0.51765 |
|  | H | -7.47293 | -2.14117 | 0.433787 |
|  | H | -5.39667 | -3.35489 | -0.0576 |
|  | H | -3.37417 | -4.59178 | -0.51775 |
|  | H | -1.23807 | -5.84207 | -1.05111 |
|  | H | -7.48477 | 2.155265 | 0.506691 |
|  | H | -8.69971 | 0.000221 | 0.670368 |
|  | C | 5.513645 | 1.281392 | 0.194227 |
|  | C | 4.810392 | 2.494534 | 0.040498 |
|  | C | 6.913729 | 1.253785 | 0.388021 |
|  | C | 3.435696 | 2.5274 | -0.12922 |
|  | C | 2.759234 | 3.762978 | -0.35913 |
|  | C | 1.398273 | 3.800392 | -0.53173 |
|  | C | 0.645984 | 5.022852 | -0.73057 |
|  | C | -0.70735 | 5.019092 | -0.72427 |
|  | C | 6.918719 | -1.16022 | 0.354235 |
|  | C | 7.598271 | 0.046995 | 0.474744 |
|  | H | 7.44577 | 2.195486 | 0.461008 |
|  | H | 5.367491 | 3.425139 | 0.017986 |
|  | H | 3.342536 | 4.676922 | -0.38877 |
|  | H | 1.200683 | 5.94543 | -0.85885 |
|  | H | -1.26848 | 5.938449 | -0.84761 |
|  | H | -3.39877 | 4.658924 | -0.35951 |
|  | H | -5.41485 | 3.396199 | 0.056324 |
|  | H | 1.231176 | -5.8355 | -1.06038 |
|  | H | 3.364066 | -4.57409 | -0.5391 |
|  | H | 5.382788 | -3.32692 | -0.08136 |
|  | H | 7.455116 | -2.10119 | 0.40008 |
|  | H | 8.671894 | 0.047096 | 0.625528 |
|  | N | 1.304695 | -1.27086 | -0.298 |
|  | N | -1.33044 | -1.27923 | -0.28061 |
|  | N | 1.299001 | 1.358794 | -0.24739 |
|  | N | -1.33558 | 1.35155 | -0.22626 |
|  | Cu | -0.01273 | 0.031418 | 0.145753 |
|  | O | 0.034259 | -0.00317 | 2.32436 |
|  | O | 0.518401 | -1.37919 | 2.809981 |
|  | H | 1.502128 | -1.20887 | 2.697521 |
|  | H | -0.85612 | 0.010415 | 2.781933 |
